# Designing Reactive Bridging O^2–^ at the Atomic Cu–O–Fe Site for Selective NH_3_ Oxidation

**DOI:** 10.1021/acscatal.2c04863

**Published:** 2022-11-29

**Authors:** Xuze Guan, Rong Han, Hiroyuki Asakura, Zhipeng Wang, Siyuan Xu, Bolun Wang, Liqun Kang, Yiyun Liu, Sushila Marlow, Tsunehiro Tanaka, Yuzheng Guo, Feng Ryan Wang

**Affiliations:** † Department of Chemical Engineering, University College London, Roberts Building, Torrington Place, LondonWC1E 7JE, U.K.; ‡ School of Electrical Engineering and Automation, Wuhan University, Wuhan430072, China; § Functional Materials Lab, Faculty of Science and Engineering, Kindai University 3-4-1, Kowakae, Higashi-Osaka, Osaka577-8502, Japan; ∥ Department of Molecular Engineering, Graduate School of Engineering, Kyoto University, Kyotodaigaku Katsura, Nishikyo-ku, Kyoto615-8510, Japan

**Keywords:** reactive O^2−^, oxidation chemistry, heterogeneous catalysis, NH_3_ emission control, single-atom catalyst

## Abstract

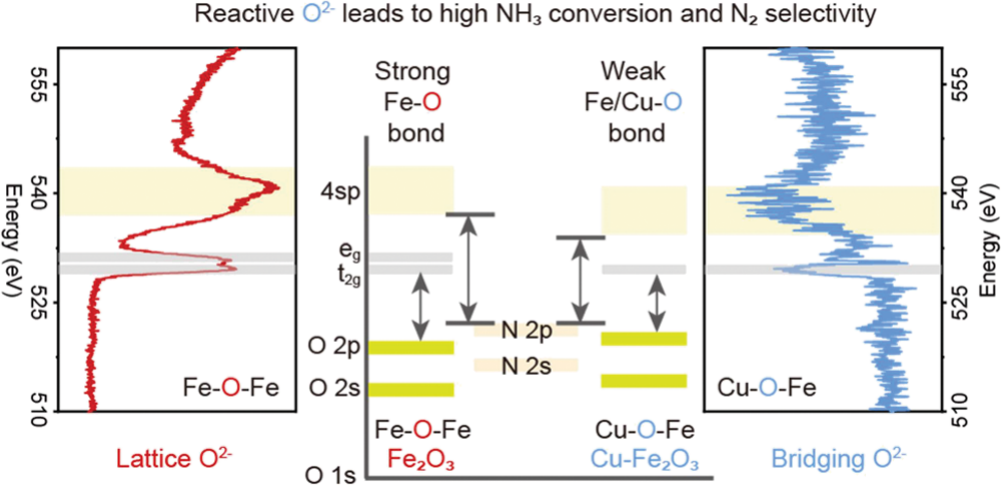

Surface oxidation chemistry involves the formation and breaking of metal–oxygen (M–O) bonds. Ideally, the M–O bonding strength determines the rate of oxygen absorption and dissociation. Here, we design reactive bridging O^2–^ species within the atomic Cu–O–Fe site to accelerate such oxidation chemistry. Using in situ X-ray absorption spectroscopy at the O K-edge and density functional theory calculations, it is found that such bridging O^2–^ has a lower antibonding orbital energy and thus weaker Cu–O/Fe–O strength. In selective NH_3_ oxidation, the weak Cu–O/Fe–O bond enables fast Cu redox for NH_3_ conversion and direct NO adsorption via Cu–O–NO to promote N–N coupling toward N_2_. As a result, 99% N_2_ selectivity at 100% conversion is achieved at 573 K, exceeding most of the reported results. This result suggests the importance to design, determine, and utilize the unique features of bridging O^2–^ in catalysis.

## 1 Introduction

What happens when a single metal atom is added onto a metal oxide surface? Taking Cu^2+^ as an example, the 3d of 4sp of Cu^2+^ will first hybridize with the conduction and valence bands of metal oxides, forming four Cu–O–M bonds.^1−4^ Due to the different energies of Cu and M valence orbitals, the resultant Cu–O and O–M bonds in the atomic site usually have less bond strength than those in bulk CuO and MO. Those bridging O^2–^ in Cu–O–M should be more reactive and can be easily taken away by reducing agents compared with lattice O^2–^. It is therefore important to probe the electronic structures of those bridging O^2–^ and rationalize their impact in catalytic reactions. In particular, for oxidation reactions such as CO and NH_3_ oxidation, the rapid O^2–^ removal and formation are the key to activate reductive reactants and molecular O_2_,^5−7^ which is ideal for the bridging O^2–^ due to its weakened bond strength.

Various nitrogen-containing pollutants (NH_3_ and NO_*x*_) have raised concerns on public health and environmental protection, which has led to increasingly strict emission standards. As one of the most promising methods for removing ammonia,^8^ the selective catalytic oxidation of NH_3_ (NH_3_-SCO) to nitrogen has received increasing attention^9,10^ and will play a crucial role in the upcoming EU7 standard. However, achieving high activity and nitrogen selectivity simultaneously remains a challenge due to the competing NO desorption pathway before its coupling reaction with NH_3_ to form N_2_, as shown below in the internal selective catalytic reduction (i-SCR) mechanism.
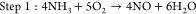
1
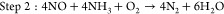
2
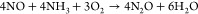
3

Having been widely accepted in NH_3_-SCO^11−16^ with transition metals, i-SCR includes two steps: First, ammonia is oxidized to NO (eq 1). Second, an N–N bond is formed when the as-prepared NO reacts with unreacted ammonia (eqs 2 and 3). The reaction rate in eqs 1–3 is defined as *r*1, *r*2, and *r*3, respectively. Based on this two-step mechanism, high selectivity toward N_2_ can only be achieved under *r*2 = *r*1 ≫ *r*3 conditions.

Eq 1 is a typical oxidation reaction promoted by the adsorption of both NH_3_ and O_2_ with immediate desorption of NO. Noble metals, such as Pt and Pd, generally favor oxygen adsorption and activation, resulting in high oxidation activity.^17,18^ However, excessive oxidation leads to low nitrogen selectivity above 300 °C in NH_3_-SCO.^19,20^ In comparison, Cu is preferred in the selective oxidation reaction due to the weak adsorption of O_2_.^3^ As a result, Cu catalysts are commercially used for the reduction of NO with NH_3_.^21−23^ For NH_3_-SCO, Cu catalysts are highly active for SCR (eq 2) but suffer from low activity in eq 1.^24,25^ Considering this, an enhanced NO formation rate and the subsequent N–N coupling to N_2_ are required for Cu catalysts to achieve a high N_2_ yield. This can be realized by modifying the reactant and intermediate adsorption behaviors by tuning the electronic structure of the Cu–O–M sites,^26,27^ especially the state of bridging O^2–^. In addition, the adsorption energy of intermediates is also changed when adsorbing on bridging O^2–^.^28−30^ In NH_3_-SCO, this means rapid O^2–^ removal/formation for NO formation and strong Cu–O–NO adsorption for N–N coupling to N_2_.

Here, we design bridging O^2–^ over atomic Cu–O–Fe to balance the rate of NO formation and N–N coupling in order to achieve high NH_3_ conversion and N_2_ selectivity. The atomic Cu sites significantly promote the N_2_ yield of Fe_2_O_3_ by 4.8 times at 573 K. The weak Cu–O–Fe bonding is determined with in situ Δ near-edge X-ray absorption fine structure (NEXAFS) and density functional theory (DFT) calculations. The special electronic states of O^2–^ of Cu–O–Fe improve the surface adsorption of in situ-formed NO by 1.18 eV, promoting the N–N coupling toward N_2_. Furthermore, 1 wt % CuO–Fe_2_O_3_ achieves 99% yield of N_2_ with a weight hour space velocity (WHSV) of 120 mL_NH3_·h^–1^·g^–1^, which surpasses most reported literature values. Thus, the ability to design and study the bridging O^2-^ of active metal sites is important to achieve the desired performance in catalysis.

## 2 Materials and Methods

### Catalyst Preparation

The copper-based catalysts were prepared by coprecipitation using copper nitrate trihydrate (Cu(NO_3_)_2_·3H_2_O), ferric nitrate nonahydrate (Fe(NO_3_)_3_·9H_2_O), and ammonium hydroxide (NH_3_·H_2_O) as starting materials. In a typical procedure, 2 g of the nitrate precursor (Fe(NO_3_)_3_·9H_2_O) and the corresponding mass of Cu(NO_3_)_2_·3H_2_O were dissolved in 15 mL of deionized water. Subsequently, the mixture was stirred for 10 min, and then NH_3_·H_2_O was added dropwise until a pH of 9 was reached under continuous stirring for 10 min. The sample was filtered and washed with deionized water and then dried at 60 °C for 12 h. The as-prepared sample was placed in a muffle furnace and calcined at 550 °C in air for 4 h. Finally, the sample was slowly cooled to room temperature in the muffle furnace, and the obtained solid was ground to powder.

By changing the loading of Cu, catalysts with different Cu aggregation states were prepared using the above method, which were atomic sites (1 wt % Cu), clusters (20 wt % Cu), and inverse catalysts (70 wt % Cu), respectively. For comparison, pure CuO and Fe_2_O_3_ were also prepared using the above method.

The 1 wt % CuO–Fe_2_O_3_ (impregnation) was prepared using the wetness impregnation method. Cu(NO_3_)_2_·3H_2_O was dissolved in deionized water and then added into Fe_2_O_3_ prepared using the above method. The sample was then dried at 60 °C for 12 h. Finally, the dried sample was calcined in air at 300 °C for 4 h at a heating rate of 5 °C/min.

### 2.1 Ex Situ Characterizations

X-ray diffraction (XRD) measurements were performed using a StadiP diffractometer from STOE with a Mo source (Kα = 0.7093165 Å). The operating voltage and current are 40 kV and 30 mA, respectively. With a resolution of 0.015° each step, the signals of 2θ in the range of 2°–40° were collected.

Scanning transmission electron microscopy (STEM) images of the samples were recorded using the JEM-ARM200CF equipped with bright field (BF) and high-angle annular dark field (HAADF) at 200 keV at the Diamond Light Source. The samples were loaded onto Au grids by sprinkling a small amount of dry sample powder.

The energy-dispersive X-ray spectroscopy (EDX) analysis of the sample was performed using the JEM-ARM200CF equipped with a large solid-angle dual EDX detector. The data were collected in the STEM illumination mode at 200 kV and corrected using special drift correction. Au TEM grids are used to avoid any Cu EDX signals from the grid. Each EDX spectrum image is 100 × 100 pixels in size, with 0.05 s exposure time per pixel.

X-ray absorption near-edge structure (XANES) and extended X-ray absorption fine structure (EXAFS) analyses of the Cu K-edge (8.979 keV) were carried out at the Diamond Light Source (UK), PETRA III DESY (Germany), and SPring-8 (Japan). The samples (≥5 wt % Cu loading) were diluted with boron nitride and pressed into a pellet with a diameter of 1.3 cm for transmission measurements. Samples with 1 wt % Cu loading were directly pressed into pellets for fluorescence measurements. A Cu foil standard was used for energy shift calibration. For the EXAFS evaluation, at least three spectra were merged to improve the signal quality.

The XAFS data were analyzed using the Demeter software package (including Athena and Artemis).^31^ Athena software was used for XANES analysis. Artemis software was used to fit the *k*^2^-weighted (CuO–Fe_2_O_3_) in real space with 3.0 Å^–1^ < *k* < 12.0 Å^–1^ and 1.0 Å < *R* < 3.3 Å. The calculated amplitude reduction factor S_0_^2^ from the EXAFS analysis of the Cu foil was 0.878, which was used as a fixed parameter for EXAFS fitting. The coordination number and bond length were calculated based on the reported structure from the Crystal open database: copper (No. 9013014) and tenorite (No. 1011148).

### 2.2 Near Ambient Pressure (NAP)-NEXAFS Spectroscopy

In situ NEXAFS experiments for 1 wt % CuO–Fe_2_O_3_ were performed at the ISISS beamline of BESSY II in Berlin (Germany). The X-ray was sourced from a bending magnet (D41) and a plane grating monochromator with an energy range from 80 to 2000 eV (soft X-ray range) and a flux of 6 × 10^10^ photons/s with 0.1 A ring current using a 111 μm slit and an 80 μm × 200 μm beam spot size. The reaction products were online-monitored using an electron impact mass spectrometer (“PRISMA,” Pfeiffer Vacuum GmbH, Asslar (Germany)) connected directly to the main experimental chamber by a leak valve. The pressure in the specimen chamber was precisely controlled (UHV or 0.1–1 mbar) by simultaneous operation of several mass flow controllers for reactive gases and a PID-controlled throttle valve for pumping gas out. Then, 100 mg of catalysts was pressed into pellets with a diameter of 6 mm. The sample pellets (6 mm diameter) were heated uniformly by an IR laser mounted on the rear part of the sample holder. Temperature control was realized by two K-type thermocouples. The NEXAFS spectra at the Cu L-edge (920–965 eV), Fe L-edge (700–740 eV), O K-edge (510–560 eV), and N K-edge (390–420 eV) were measured in either the total electron yield (TEY) mode or the Auger electron yield (AEY) mode.

### 2.3 In Situ XANES

In situ XANES experiments for 1 wt % CuO–Fe_2_O_3_ were performed at SPring-8 in Japan. In the experiment, 1 wt % CuO–Fe_2_O_3_ was pressed into a pellet and measured at room temperature, 573 and 673 K. XANES spectra of each gas composition were recorded between 6770 and 8160 eV in the transmission mode for Fe K edge with a Si(111) crystal monochromator. The Cu K edge XANES spectra were measured in the fluorescence mode with a Si(111) crystal monochromator. The spectra processing was also performed with Athena.

### 2.4 In Situ Diffuse Reflectance Infrared Fourier Transform Spectroscopy (DRIFTS)

The DRIFTS analysis was performed with a PerkinElmer Frontier FTIR spectrometer. For this analysis, 37–57 mg of sample was made into self-supporting wafers. To remove surface contamination, the sample was heated up in 7% O_2_/He from 30 to 350 °C with a ramp of 10 °C/min. During this ramp, the spectra were recorded as background at different temperatures. After holding at 350 °C for 30 min, the sample was cooled to 30 °C and purged in He until complete O_2_ removal. The sample was then exposed to 500 ppm of NO/He for 30 min during which the spectra were recorded. Then, the sample was purged with He for 10–15 min while recording the spectra. After purging, the sample was heated up in a He environment from 30 to 350 °C with a ramp of 10 °C/min.

The spectra were recorded in the range of 4400–500 cm^–1^ with a resolution of 2 cm^–1^. The spectra at 30 °C in He were used as background for NO adsorption and He purge data. The spectra recorded in O_2_/He were used as background for NO-TPD. All spectra were normalized by the sample weight.

### 2.5 Catalytic Performance Measurement

The ammonia selective catalytic oxidation was evaluated in a fixed-bed flow reactor. The composition and flow rate of the inlet gas mixture were set by the mass flow controller. The typical reaction gas composition was 5000 ppm of NH_3_, 5 vol % O_2_, and balance He. The flow rate of the mixed gas was 100 mL/min. Typically, 50 mg of catalyst was placed in the reaction tube, and the product was detected with the quadrupole mass spectrometer (MS) quantitative gas analyzer (Hiden Analytical, UK). The reaction was studied in the temperature range of 473 to 673 K. After reaching the steady state at each reaction temperature, the reaction was maintained for at least 30 min to measure the MS signals of the reactants (NH_3_ and O_2_) and products (N_2_, N_2_O, and NO).

The stability test of 1 wt % CuO–Fe_2_O_3_ was conducted under 473 K for 100 h (WHSV = 120 mL_NH3_ × h^–1^ × g^–1^).

### 2.6 DFT Calculations

The spin-polarized DFT + U calculations were performed through the calculation software QuantumATK.^32^ All the geometries were fully optimized by using the Perdew–Burke–Ernzerhof functional. All the electronic structures were calculated by using the Heyd–Scuseria–Ernzerhof hybrid functional.^33^ The values of *U* were carefully chosen according to relevant paper, which are 7 and 4 eV for Cu atoms and Fe atoms, respectively. The Brillouin zone was sampled using a 9 × 9 × 9 Monkhorst–Pack^34^*k*-point mesh and 520 Hartrees cutoff energy for the primitive cell lattice optimization, while 1 × 1 × 1 *k*-points are employed for subsequent adsorption calculations. During the optimization, the convergence criteria were set to 0.03 eV/A and 10^–5^ eV for force and energy, respectively. We chose the (0001) surfaces of Fe_2_O_3_ and (111) surfaces of CuO owing to their lower surface energy and greater stability.

The adsorption energy per molecule was calculated as follows:
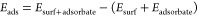
where *E*_surf + adsorbate_ is the total energy of the whole system which includes the gas molecule and the surface slab structure. *E*_surf_ and *E*_adsorbate_ are the energy of a sole surface slab and an isolated gas molecule, respectively. According to the equation, the negative adsorption energy indicates the existence of adsorption while the positive one means no evident adsorption interactions.

## 3 Results and Discussion

### 3.1 Distribution and Structure of Copper over Metal Oxides

**Figure 1:**
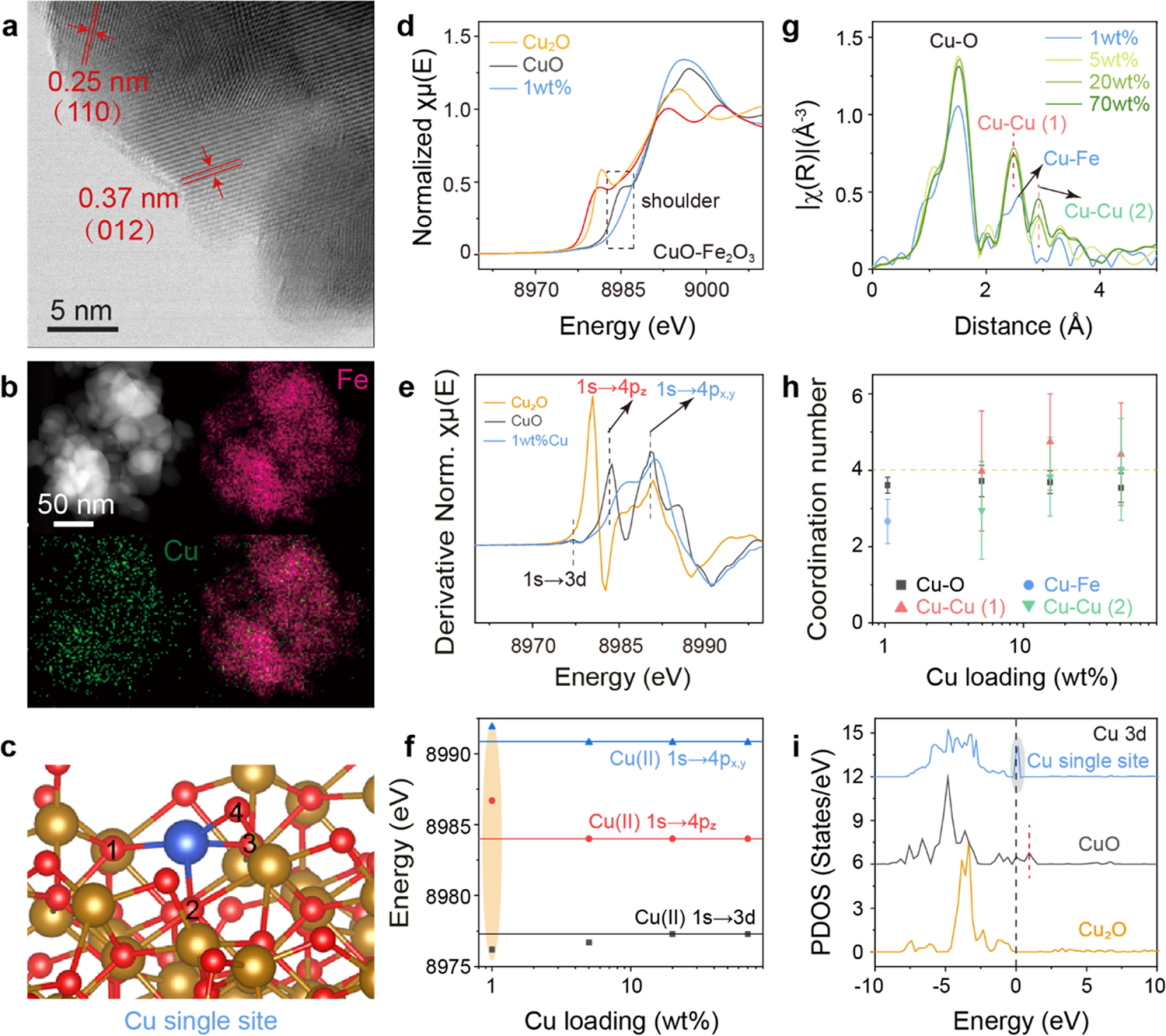
Distribution and electronic structure of the atomic Cu(II) site. (a) BF-STEM image for 1 wt % Cu on Fe_2_O_3_. (b) EDX mapping of 1 wt % Cu on Fe_2_O_3_. (c) DFT-calculated model of the structure of a Cu single site. (d) Cu K-edge XANES spectra of CuO–Fe_2_O_3_ with 1 wt % Cu loading and Cu_2_O and CuO standards. (e) First derivative of Cu K-edge XANES spectra of CuO–Fe_2_O_3_ with 1 wt % Cu loading and Cu_2_O and CuO standards. (f) Peak position of 1s → 3d and 1s → 4p electron transitions in the first derivative of Cu K-edge XANES spectra for CuO–Fe_2_O_3_ catalysts. (g) EXAFS spectra of CuO–Fe_2_O_3_ with various Cu loading. (h) Coordination number (C.N.) of Cu–O, Cu–Fe, Cu–Cu (1), and Cu–Cu (2) in CuO–Fe_2_O_3_ as a function of Cu loading. (i) Projected density of states (PDOS) of the Cu-d orbit in Cu_2_O, CuO, and Cu single site over Fe_2_O_3_.

Fe_2_O_3_ nanoparticles with an average size around 45 nm are obtained via precipitation (Figure S1). Coprecipitating with Cu does not change the morphology of the particles (Figures S2 and S3). The lattice fringes of Fe_2_O_3_(110) and Fe_2_O_3_(012) facets are observed in the BF-STEM images (Figures 1a). The presence of Cu is confirmed in the EDX spectrum, which shows Cu Kα emission at 8.05 keV even at only 1 wt % loading (Figure S4).

Cu is uniformly dispersed on iron oxide support, as shown in the homogeneous Cu map alongside that of Fe (Figure 1b). The ksp of Fe(OH)_3_ is 1.1 × 10^–36^, while the ksp of Cu(OH)_2_ is 4.8 × 10^–20^. With 1 wt % CuO loading, the molar ratio of Fe and Cu is 98.6. Although Fe is much more than Cu, Cu starts to precipitate after most of Fe has precipitated. Therefore, most of the Cu sites in 1 wt % CuO–Fe_2_O_3_ prepared by the coprecipitation method are on the surface of the catalyst. DFT calculations were performed to identify stable Cu structures on the (0001) surface of Fe_2_O_3_ (Figure 1c). The Cu atom is coordinated with four O atoms, which is confirmed by the EXAFS results. The EXAFS analysis shows no Cu–Cu scattering for 1 wt % CuO–Fe_2_O_3_ (Figure 1g,h and fitting results in Figure S5 and Table S1), suggesting the formation of atomic Cu–O–Fe; the detailed discussion is provided in Supplemental Note 1 and Figure S6.

The Cu(II) oxidation state is confirmed with XANES, which shows the 1s → 3d transition for the Cu(II) d^9^ configuration between 8976.2 and 8977.3 eV (Figure 1d,e). The absorption energy of 1s → 3d transitions decreases from 8977.2 eV for pure CuO to 8976.2 eV for 1 wt % CuO–Fe_2_O_3_, whereas that of the 1s → 4p_z_ transition increases by 2.7 eV from CuO for 1 wt % CuO–Fe_2_O_3_ (Figure 1f and Table S2). The calculated PDOS of Cu atoms further confirms that the unoccupied Cu 3d state of the Cu single site over Fe_2_O_3_ is located slightly above the Fermi level, which is lower than that of CuO (Figure 1i). A Bader charge analysis is performed to analyze the charge transfer between Cu(II) sites and Fe^3+^ on the Fe_2_O_3_ (0001) surface. Compared with pure copper oxide, atomic Cu sites on Fe_2_O_3_ are more positively charged (Tables S3 and S4). According to the literature, such reduced 1s → 3d and increased 1s → 4p_z_ transitions suggest the formation of atomic sites with strong interactions with the support that increase the energy of the lowest unoccupied molecular orbital (LUMO) and reduce the energy of the highest occupied molecular orbital (HOMO).^3^ The Cu(II) site has the 3d^9^ configuration, and the energy levels of the occupied 3d orbitals and unoccupied 4p orbitals are considered as HOMO and LUMO, respectively. The 1s → 3d transition toward the half-empty orbital of Cu(II) can reflect the HOMO of Cu(II). In addition, the 1s → 4p_z_ transition of 1 wt % CuO–Fe_2_O_3_ is very close to the 1s → 4p_*xy*_ peaks (Figure 1e blue), indicating that the symmetry of Cu(II) has changed.^35^ In comparison, CuO (Figure 1e black) and CuO clusters over Fe_2_O_3_ (Figure S7) have been separated into 1s → 4p_z_ and 1s → 4p_*xy*_ peaks, which can be explained via the Jahn–Teller effect.^36^ We hypothesize that in 1 wt % CuO–Fe_2_O_3_, Cu^2+^ replaces the position of lattice Fe^3+^, forming Cu^2+^–O–Fe^3+^ that changes the electronic structures of both Cu^2+^ and O^2–^.

To further prove this, 1 wt % CuO–Fe_2_O_3_ prepared by impregnation has Cu species out of the lattice, showing separated 1s → 4p_z_ and 1s → 4p_*xy*_ peaks, which are different from that of the coprecipitated Cu. As shown in Figure S8, the 1s → 4p_z_ peak of Cu in Cu–Fe_2_O_3_ prepared by coprecipitation becomes a shoulder. This is due to the fact that some Cu^2+^ are in the Fe^3+^ location with a reduced Jahn–Teller effect. Although with the reduced Jahn–Teller effect, the Cu single sites are still in an elongated octahedral alignment. The two O in the *z-*axis are difficult to observe and fit in the R-space. The EXAFS spectrum of the impregnated sample is similar to that of the CuO standard, which is also different with coprecipitated samples (Figure S8). This suggests the possibility of Cu agglomeration on the surface of the impregnated samples, which leads to a stronger Jahn–Teller effect.

Combining the XANES and EXAFS results, we confirm that atomic Cu sites with a lower 3d^9^ energy level are formed on Fe_2_O_3_ at 1 wt % Cu loading. Increasing the Cu loading to 5 wt % or beyond forms CuO clusters (Figure S9). The diffraction features of CuO can be observed with 20 wt % CuO–Fe_2_O_3_ (Figure S10), which is in good agreement with the BF-STEM image. These surface CuO clusters have similar d states as the CuO standard (Figure 1e, and Table S2) and are different from atomic Cu–O–Fe sites.

### 3.2 Catalytic Activity on NH_3_-SCO

**Figure 2:**
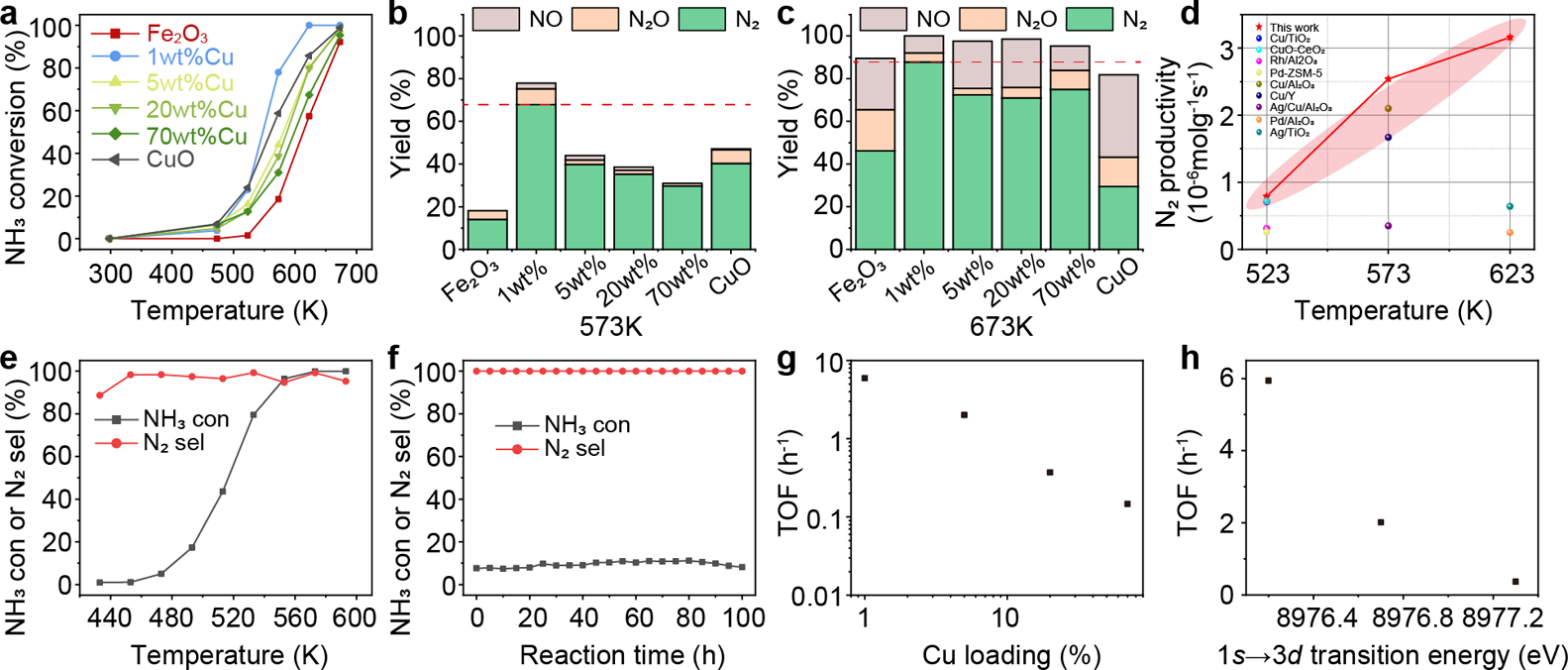
Catalytic performance of Cu species in NH_3_-SCO. (a) NH_3_ conversion as a function of temperature for CuO–Fe_2_O_3_ catalysts. The catalytic profile and selectivity versus conversion plots are given in Figure S13. (b) Yields of N_2_, N_2_O, and NO for CuO–Fe_2_O_3_ catalysts at 573 K. (c) Yields of N_2_, N_2_O, and NO for CuO–Fe_2_O_3_ catalysts at 673 K. Reaction conditions: 50 mg of catalyst, 5000 ppm of NH_3_, 5% O_2_ balanced in He, a gas flow of 100 mL/min, and WHSV = 600 mL_NH3_·h^–1^·g^–1^. (d) Comparison of N_2_ productivity for 1 wt % CuO–Fe_2_O_3_ at a WHSV of 600 mL_NH3_·h^–1^·g^–1^ with other catalysts. (e) NH_3_ conversion and N_2_ selectivity as a function of temperature for the 1 wt % CuO–Fe_2_O_3_ catalyst. Reaction conditions: 50 mg of catalyst, 1000 ppm of NH_3_, 5% O_2_ balanced in He, a gas flow of 100 mL/min, and WHSV = 120 mL_NH3_·h^–1^·g^–1^. (f) Stability test of 1 wt % CuO–Fe_2_O_3_ at a WHSV of 120 mL_NH3_·h^–1^·g^–1^ under 473 K. (g) TOF as a function of Cu loading. (h) TOF as a function of 1s → 3d transition energy of Cu.

We compare the catalytic behavior of atomic Cu–O–Fe with that of the clusters. For pure oxides, a shift from high N_2_ selectivity to high NO selectivity is observed along with an increase in NH_3_ conversion (Figure S11). This trend is consistent with the i-SCR mechanism.^37,38^ Atomic Cu–O–Fe improves the NH_3_ conversion on Fe_2_O_3_ surfaces (Figures 2a and S12). With the lowest 1s → 3d transition energy, atomic Cu–O–Fe achieves 2 times higher NH_3_ conversion compared with CuO clusters at 573 K and keeps 88% N_2_ selectivity at 673 K with 5000 ppm of inlet NH_3_ (Figure 2b,c). In comparison, the catalytic performance of physically mixed 1 wt % CuO + 99 wt % Fe_2_O_3_ is not different from that of pure Fe_2_O_3_ (Figure S13). 1 wt % CuO–Fe_2_O_3_ prepared by impregnation gave less NH_3_ conversion and N_2_ selectivity compared to the coprecipitated 1 wt % CuO–Fe_2_O_3_ (Figure S8c), suggesting that atomic Cu–O–Fe in the lattice promotes both oxidation ability and NO adsorption and thus improving the N_2_ yield. Compared to other catalyst systems in the literature,^13,39−44^ the atomic Cu–O–Fe catalyst achieved the highest N_2_ productivity of Cu-based catalysts, even higher than some noble metal catalysts between 523 and 623 K (Figure 2d). Under realistic NH_3_ slip conditions (1000 ppm of NH_3_ and a WHSV of 120 mL_NH3_·h^–1^·g^–1^), 100% NH_3_ conversion and 99% N_2_ selectivity are achieved at 573 K (Figure 2e), suppressing most of the reported catalysts (Table S5). The atomic Cu–O–Fe catalyst also offers good stability for at least 100 h (Figure 2f) under a high WHSV, showing its potential toward replacing expensive Pt catalysts.

As Fe_2_O_3_ provides negligible NH_3_ conversion at 473 K, the Cu-based turnover frequency (TOF) was then calculated. In general, the TOF reduces as the loading of Cu increases (Figure 2g). The HOMO level (1s → 3d), which reflects the interaction between Cu and supports, is negatively correlated with TOF in the NH_3_-SCO activity (Figure 2h). The atomic Cu–O–Fe with the lowest HOMO has the highest TOF of 5.9 h^–1^, which is 56 and 16 times higher than that of pure CuO and CuO clusters (20 wt %) over Fe_2_O_3_. In addition to the different chemical environments of Cu, the agglomeration of CuO clusters at higher Cu loadings reduces the amount of accessible Cu, leading to the decrease of TOF. These results confirm our hypothesis that the catalytic performance can be modified significantly by forming atomic Cu–O–M sites.

### 3.3 Determination of Bridging O^2–^ in Cu–O–Fe and Its Impact on NH_3_ Conversion

Loading just 1 wt % of Cu(II) onto Fe_2_O_3_ leads to 15 times higher conversion at 523 K (22.9 vs 1.5%), suggesting that atomic Cu–O–Fe is the major active species for NH_3_ oxidation. The conversion of NH_3_ to NO is the first step and the rate-determining step in NH_3_ oxidation, which involves the Cu^+^/Cu^2+^ redox and bridging O^2–^ removal/formation. The literature suggested that oxides with weak metal–oxygen bonds exhibit good redox properties and thus have higher rates of NO formation.^11^ NAP-NEXAFS and in situ XAFS experiments are performed to identify bridging O^2–^ and its dynamics under NH_3_ oxidation conditions.

**Figure 3:**
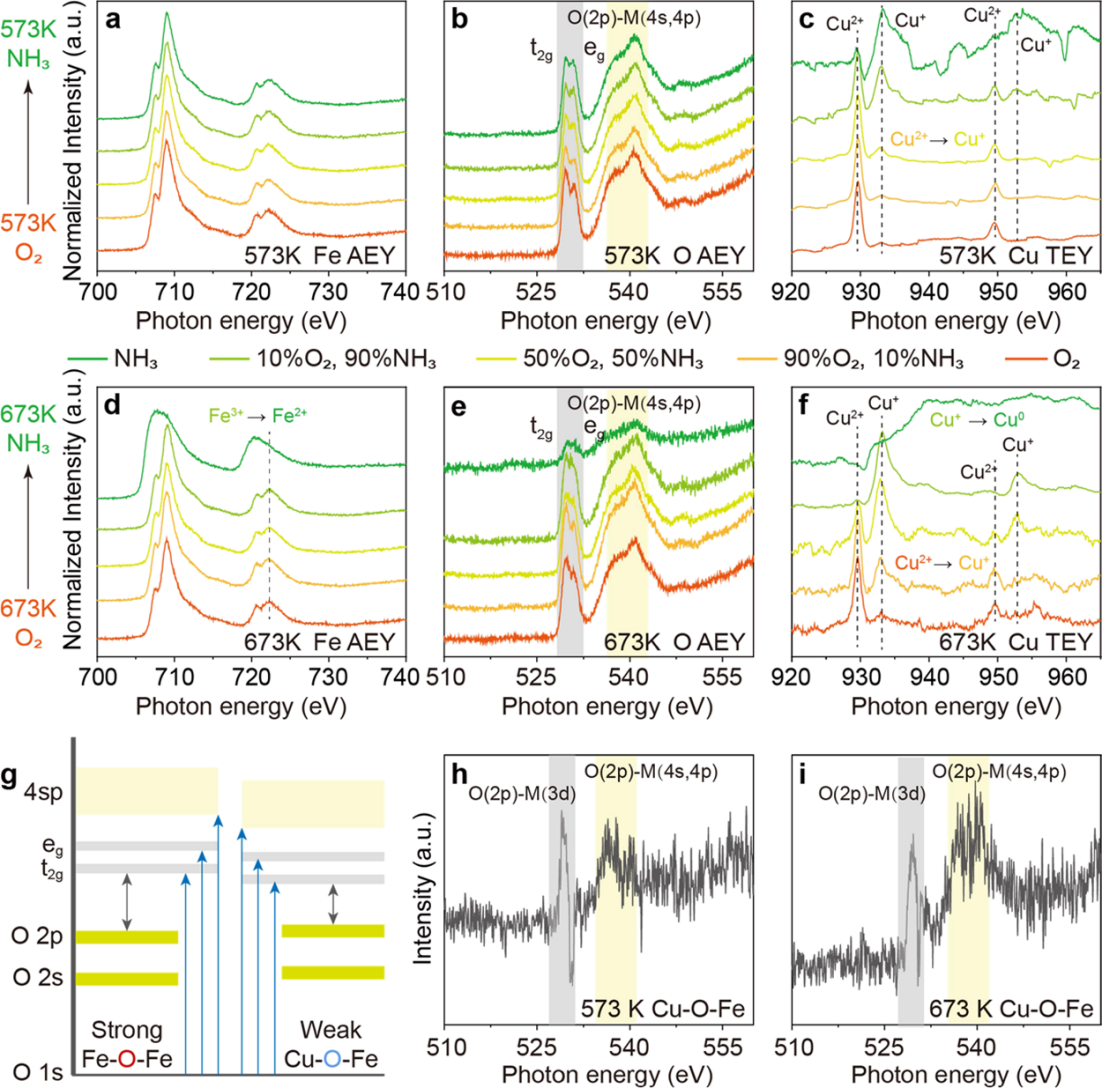
NAP-NEXAFS study of the redox behavior of 1 wt % Cu over an Fe_2_O_3_ support. (a–c) NEXAFS spectra of 1 wt % CuO–Fe_2_O_3_ under various gas conditions at 573 K. (a) Fe L-edge (AEY mode), (b) O K-edge (AEY mode), and (c) Cu L-edge (TEY mode) of 1 wt % CuO–Fe_2_O_3_ at 573 K. (d–f) NEXAFS spectra of 1 wt % CuO–Fe_2_O_3_ under various gas conditions at 673 K. (d) Fe L-edge (AEY mode), (e) O K-edge (AEY mode), and (f) Cu L-edge (TEY mode) of 1 wt % CuO–Fe_2_O_3_ at 673 K. (g) Interpretation of the oxygen K-edge XAS spectrum of O in Fe–O–Fe and bridging O in Cu–O–Fe. (h,i) ΔNEXAFS of the O K-edge of 1 wt % CuO–Fe_2_O_3_. (h) ΔNEXAFS of the O K-edge between pure O_2_ and pure NH_3_ condition at 573 K. (i) ΔNEXAFS of the O K-edge between pure O_2_ and 90% NH_3_ condition at 673 K.

The pre-edge of the O K-edge is separated into two peaks (1s to Fe 3d t_2g_ and e_g_ and Cu 3d e_g_, ligand to metal charge transfer) due to the ligand-field splitting, which reflects the transitions to antibonding O 2p states hybridized with the 3d metal states (Figure 3g).^45^ For pure α-Fe_2_O_3_, the ratio of 1s to t_2g_ and 1s to e_g_ peak is about 1:1.^46^ When atomic Cu is loaded over Fe_2_O_3_, the peak of 1s to t_2g_ (529.7 eV) becomes slightly higher than the peak of 1s to e_g_ (531.0 eV), revealing the contribution from additional bridging O^2–^ (Cu–O–Fe). Such an influence is less when Cu^2+^ is reduced due to the change of its 3d orbital geometry (Figure 3b,e) and less Cu–O bonds (the Cu–O coordination numbers reduce from 3.28 to 2.40 when switching from O_2_ to NH_3_, Table S6). The decrease in the O K-edge spectra occurs along with the reduction in the Cu L_3_-edge spectra, confirming the removal of O in Cu–O–Fe upon Cu reduction. The O K-edge ΔNEXAFS between pure O_2_ and pure NH_3_ condition at 573 K and ΔNEXAFS between pure O_2_ and 90% NH_3_ condition at 673 K reveal the spectroscopy feature of the reactive bridging O^2–^ that can be taken away by NH_3_, with the major 1s to 2p–3d transition pre-edge at 529.4 eV (Figure 3h,i). This is a direct observation of the metal/support interface. For the O in Cu–O–Cu, the energy position of O 2p–metal 3d is reported to be similar to O in Fe_2_O_3._^47^ Compared with the O in Fe–O–Fe, the reactive O^2–^ in Cu–O–Fe has lower 2p–3d and 2p–4sp energies by 0.3 and 0.7 eV (Figure 3b,e and h,i as comparison), suggesting weaker Fe/Cu–O bonds (Figure 3g). The lower 2p–3d energy is consistent with the decreased 3d^9^ energy of the atomic Cu sites. Such weak Fe/Cu–O bonds explain the reactivity of bridging O^2–^ that can be easily taken away by NH_3_. Along with the removal of O^2–^ is the reduction of Cu^2+^ to Cu^+^, as shown in the corresponding Cu L_3_-edge NAP-NEXAFS spectra (Figure 3c,f). The simultaneous O^2–^ removal and Cu^2+^ reduction start from 10% O_2_ + 90% NH_3_, 50% O_2_ + 50% NH_3_, and 50% O_2_ + 50% NH_3_ at 473, 573, and 673 K, respectively (Figures S14 and 3). At 573 K, only a trace amount of Cu^2+^ exists under pure NH_3_ (Figure 3c). At 673 K, all the Cu^2+/+^ is reduced to Cu^0^ with pure NH_3_ and even surface Fe^3+^ is reduced to Fe^2+^ (Figure 3d,f; the bulk is still Fe^3+^ as seen in Figure S15). Such a process takes away both bridging O^2–^ in Cu–O–Fe and lattice O^2–^ in Fe–O–Fe, resulting in almost complete removal of surface O (Figure 3e green).

**Figure 4:**
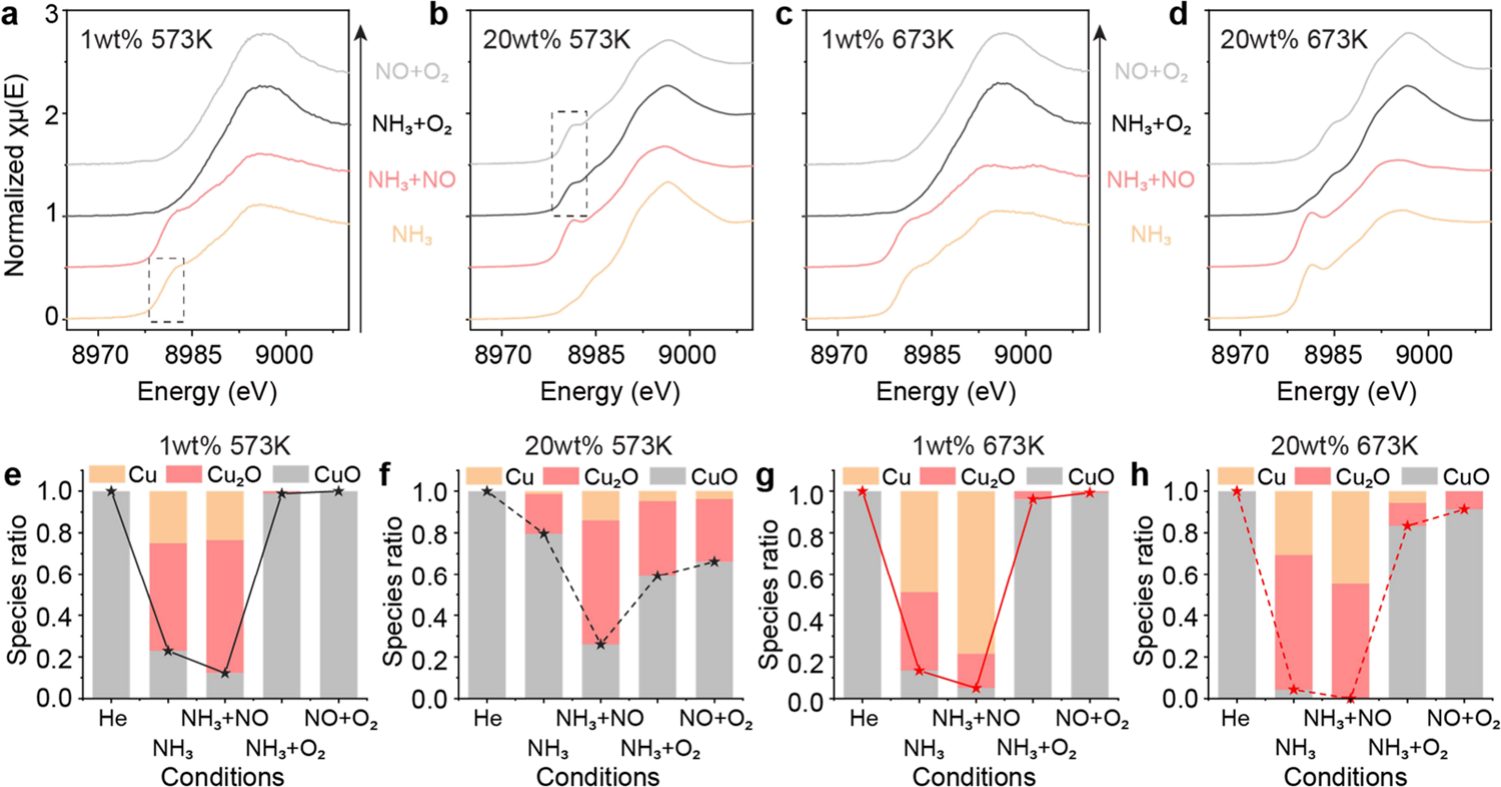
In situ XAFS study of CuO–Fe_2_O_3_ catalysts. (a,b) In situ XANES spectra of CuO–Fe_2_O_3_ catalysts with (a) 1 wt % Cu and (b) 20 wt % Cu loading at 573 K under various gas conditions. (c,d) In situ XANES spectra of CuO–Fe_2_O_3_ catalysts with (c) 1 wt % Cu and (d) 20 wt % Cu loading at 673 K under various gas conditions. (e,f) Cu speciation of CuO–Fe_2_O_3_ catalysts with (e) 1 wt % Cu and (f) 20 wt % Cu loading under He, NH_3_, NH_3_ + NO, NH_3_ + O_2_, and NO + O_2_ conditions at 573 K. (g,h) Cu speciation of CuO–Fe_2_O_3_ catalysts with (g) 1 wt % Cu and (h) 20 wt % Cu loading under He, NH_3_, NH_3_ + NO, NH_3_ + O_2_, and NO + O_2_ conditions at 673 K.

We further compare the redox behaviors of atomic Cu sites and CuO clusters over Fe_2_O_3_ by using the in situ XANES study at the Cu K-edge. The NH_3_-SCO reaction is carried out under oxidation conditions, and the oxidation state of Cu remains at Cu^2+^. Compared with the CuO clusters, atomic Cu–O–Fe sites are more easily reduced at 573 K under NH_3_ conditions (Figure 4a,e), with Cu–O coordination numbers decreasing from 3.28 to 2.40. This marks the removal of bridging O^2–^ of Cu–O–Fe. The significant reduction of Cu in 20 wt % Fe_2_O_3_ can be observed in NH_3_ + NO, which is a more reducing condition (Figure 4b,f). By switching from reduction conditions to oxidation conditions, atomic Cu–O–Fe can be fully oxidized under NH_3_ + O_2_ (Figure 4a). In comparison, Cu^+^ exists in CuO clusters even in NO + O_2_ at 573 K (Figure 4b). At 673 K, the reduction behaviors of the two types of Cu are similar (Figure 4c,d,g,h). After switching from the reducing atmosphere to the oxidizing atmosphere of NH_3_ + O_2_, almost all the atomic Cu are oxidized to Cu^2+^ (Figure 4c,g), with the formation of bridging O^2–^ (Cu–O C.N. increases to 3.88), while the CuO cluster still has a small amount of Cu^+^ which cannot be oxidized in NH_3_ + O_2_ (Figure 4d,h).

### 3.4 Promotion of N_2_ Selectivity over Bridging O^2–^

**Figure 5:**
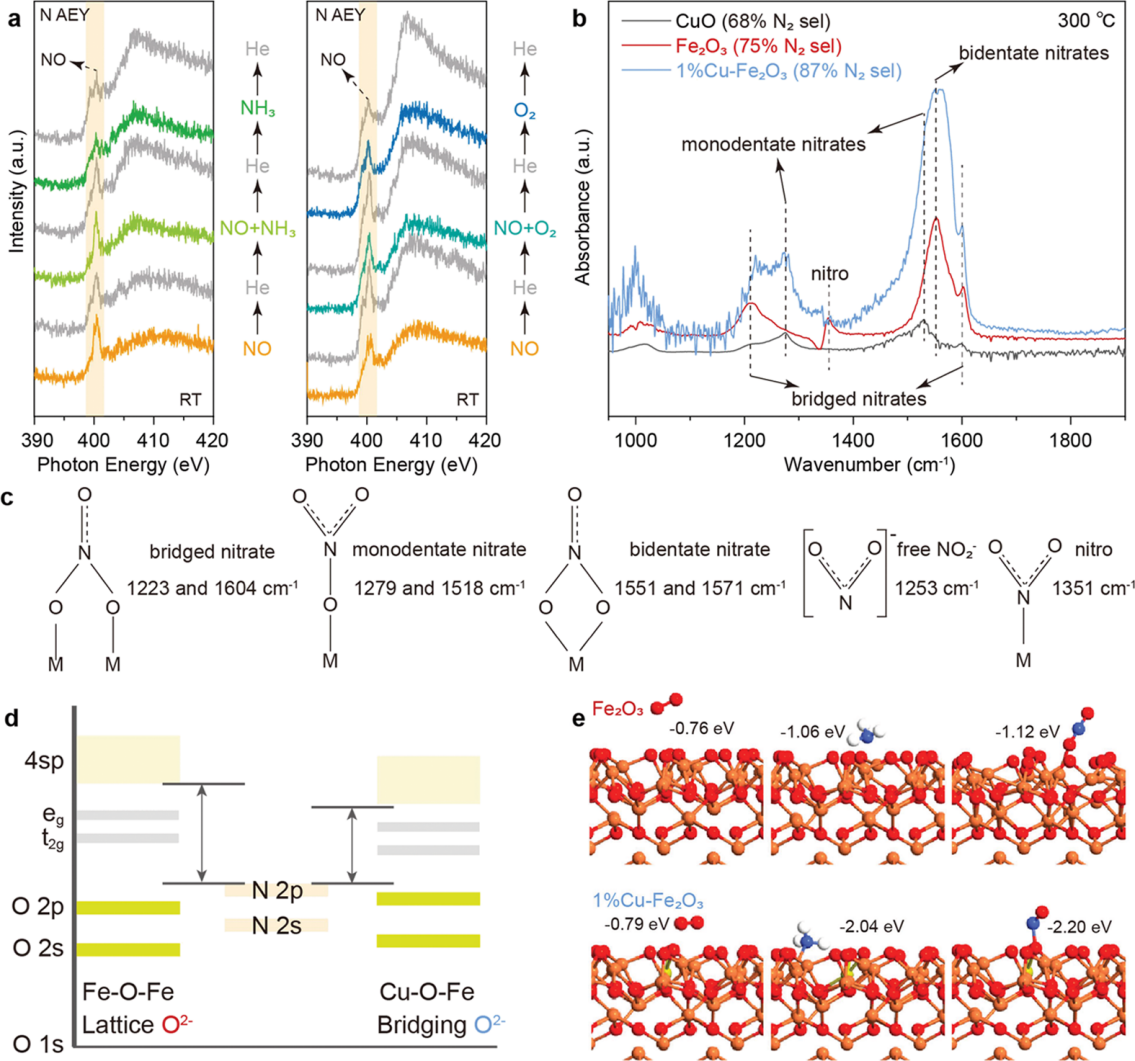
NAP-NEXAFS study, in situ DRIFTS study, and DFT calculations of 1 wt % CuO–Fe_2_O_3_. (a) NEXAFS spectra of the N K-edge (AEY mode) of 1 wt % CuO–Fe_2_O_3_ under NO to NH_3_ (left) and NO to O_2_ (right) gas atmospheres (0.3 mbar) at 298 K. The AEY mode measures the surface-adsorbed species. (b) In situ DRIFTS spectra of NO desorption in He on 1 wt % CuO–Fe_2_O_3_ (blue), Fe_2_O_3_ (red), and CuO (black) at 300 °C after the catalyst was exposed to a flow of 500 ppm of NO for 30 min at 30 °C. DRIFTS spectra were normalized by the sample weight. (c) Possible structures of surface NO_*x*_ species (M = metal ion) formed during NO desorption. (d) Interpretation of the energy matching of N in NO with O in Fe–O–Fe and bridging O in Cu–O–Fe. (e) DFT calculations of O_2_ (left), NH_3_ (middle), and NO (right) adsorption over Fe_2_O_3_ and 1 wt % CuO–Fe_2_O_3_. Cu atoms are shown in yellow, Fe atoms in orange, O atoms in red, H atoms in white, and N atoms in blue. The detailed DFT calculations are shown in Figures S22–S27.

The atomic Cu–O–Fe sites also clearly promote N–N coupling toward N_2_ formation, which is the second step of NH_3_-SCO. This indicates that the as-formed NO chooses to react with the trace amount of surface NH_3_ instead of desorbing. The PDOS calculation of the O p orbital further confirms that the unoccupied O 2p state of O in Cu–O–Fe is lower than that of O in Fe–O–Fe (Figure S16). We hypothesize that the lower 2p–4sp energy of Cu–O–Fe has a better energy match toward the nitrogen lone pair in NO (Figure 5d), contributing to the enhanced adsorption of NO, which leads to a better i-SCR rate (*r*2). The detailed discussion is provided in the Supplemental Note 2. This is investigated via a series of in situ characterization techniques and DFT calculations below.

The NAP-NEXAFS experiments confirm that the adsorption of NO is stronger than that of NH_3_ and O_2_ on 1 wt % CuO–Fe_2_O_3_. As shown in the N K-edge NAP-NEXAFS spectra, the surface-adsorbed NO (400.3 eV, Figure S17) cannot be removed by NH_3_ and O_2_ at room temperature for 1 wt % CuO–Fe_2_O_3_ (Figure 5a). In comparison, oxygen can be replaced by ammonia. On changing from O_2_ to NH_3_ atmosphere, the surface-adsorbed O_2_ over 1 wt % CuO–Fe_2_O_3_ gradually weakens, and the surface-adsorbed NH_3_ signal increases (402.1 eV, Figure S18) until the surface O_2_ is completely replaced with NH_3_.

The contribution of atomic Cu–O–Fe sites is further studied by in situ DRIFTS. At room temperature, NO is first adsorbed as bridged nitrate (1223 and 1604 cm^–1^, Figure S19) on the surface of Fe_2_O_3_ and 1 wt % CuO–Fe_2_O_3_, and then bridged nitrate is gradually transformed into monodentate nitrate at 1279 and 1518 cm^–1^.^48,49^ This phenomenon is more obvious on the surface of 1 wt % CuO–Fe_2_O_3_. An additional −OH group (3600–3800 cm^–1^) is formed over the 1 wt % CuO–Fe_2_O_3_ surface. The possible reason is that Cu^2+^ replaces Fe^3+^, so there will be more H^+^ to neutralize the charge, leading to the high intensity of the −OH group.^50,51^ The OH group over catalysts is removed under 300 °C (Figure S20). As the temperature rises, the monodentate nitrate on the Fe_2_O_3_ surface is converted into bridged nitrate, bidentate nitrate (1551 and 1571 cm^–1^), and free NO_2_^–^ ions^52^ (1253 cm^–1^) (Figure S21). Free NO_2_^–^ will then lead to the formation of surface nitro species (1351 cm^–1^) (Figure S21),^53^ which are inactive in the SCR reaction due to their structure and thermal stability.^54^ In comparison, nitro species are not seen on the surface of CuO and 1 wt % CuO–Fe_2_O_3_. The monodentate nitrate remains stable over CuO and 1 wt % CuO–Fe_2_O_3_ even at 300 °C. Compared with CuO, 1 wt % CuO–Fe_2_O_3_ has 10 times higher monodentate nitrate features, suggesting the promotion by atomic Cu–O–Fe sites. We assume that the catalyst reflectance and the (catalyst + adsorbate) reflectance of Cu–Fe_2_O_3_ and Fe_2_O_3_ are similar. Ideally, the DRIFTS intensity that is linear with the adsorbate surface concentration would be preferred. At the same temperature, the NO adsorption over 1 wt % CuO–Fe_2_O_3_ forms 3.3 times higher features for bidentate nitrate species^55,56^ at 1551 and 1571 cm^–1^ and 2.7 times higher bridged nitrate^57^ features at 1223 and 1604 cm^–1^ than Fe_2_O_3_ (Figure 5b,c). Therefore, the atomic Cu–O–Fe sites increase the surface NO density, prevent the formation of inactive nitro species, and improve the stability of the surface monodentate nitrate, which is the most active nitrate species in the SCR reaction.^58^

The Fe_2_O_3_ (0001) surface is reported to dominate under natural conditions,^59^ and therefore, the structure of the α-Fe_2_O_3_ (0001) plane and its interaction with adsorbents have been calculated. The DFT calculations compare the adsorption energy of each molecule over pure CuO (111) and Fe_2_O_3_ (0001) and Cu over a Fe_2_O_3_ (0001) surface. At the CuO (111) and Fe_2_O_3_ (0001) surfaces, the adsorption energies of NO are −1.02 and −1.12 eV. Adding one Cu atom to Fe_2_O_3_ increases the NO adsorption to −2.20 eV (Figures 5e and S22–S27 and Table S7), which is consistent with the adsorption results. The NO is absorbed on the bridging O^2–^ (as shown in Figure 1c, O marked with the number 4) of Cu-modified Fe_2_O_3_. The Bader charge analysis gives the charge of the bridging O^2–^ (4) site of 0.66 e for the Cu-modified Fe_2_O_3_ (0001) surface (Table S3), which is much lower than that of CuO (0.85 e, Table S4). After adsorption of NO, the electrons at O^2–^ (4) move toward NO (Table S8), which could be the reason for the strong NO adsorption via N–O.

### 3.5 Discussion

NAP-NEXAFS confirms that O^2–^ in Cu–O–Fe has a lower antibonding energy than O^2–^ in Fe–O–Fe, suggesting weaker Fe/Cu–O bonds. As a result, the atomic Cu–O–Fe site shows better bridging O^2–^ removal performance, which increases the NH_3_ conversion from 0 to 4% and 18 to 78% at 473 and 573 K, respectively, in comparison with lattice O^2–^ from pure Fe_2_O_3_. The in situ XAFS study indicates that the atomic Cu–O–Fe site shows better O^2–^ removal/formation and Cu^2+^/Cu^+^ redox performance than CuO clusters due to the highly active bridging O^2–^ in atomic Cu–O–Fe and thus has a better ability to oxidize NH_3_ to NO in the first step (eq 1). Therefore, the weakened Cu–O–Fe bonding forms reactive O^2–^ that enables Cu redox, leading to a higher NH_3_ oxidation activity compared with CuO clusters.

With a lower O antibonding orbital energy, we speculate that the O in Cu–O–Fe has better energy match with N in NO, leading to strong NO adsorption. In situ DRIFTS confirms the strong adsorption of nitrate species over 1 wt % CuO–Fe_2_O_3_, which suggests that the bridging O^2–^ in Cu–O–Fe offers unique NO adsorption via O–N. This is different from lattice O^2–^ in CuO (Cu–O–Cu) and Fe_2_O_3_ (Fe–O–Fe). Therefore, the reactive bridging O^2–^ not only promotes the Cu^2+^/Cu^+^ redox for NH_3_ conversion but also enhances NO adsorption on the catalyst surface for subsequent N–N coupling, which explains the 24 and 28% of N_2_ selectivity increase from pure Fe_2_O_3_ at 573 and 673 K, respectively (Figure 2b,c).

## 4 Conclusions

Our study highlights the importance of bridging O^2–^ in catalysis at the atomic Cu–O–Fe site. Such reactive O^2–^ stems from the weak bonding with Cu and Fe, as confirmed in the O K-edge ΔNEXAFS and DFT calculations. As a result, the reactive O^2–^ can be easily taken away by NH_3_, promoting the rapid Cu+/Cu^2+^ redox and NH_3_ conversion. The reduced 2p–3d antibonding orbital energy at the Cu–O–Fe site also leads to strong NO adsorption and acceleration of the N–N coupling to N_2_ as the final product. Thus, the Cu–O–Fe sites show a 16 times higher activity than CuO clusters. The bridging O^2–^ in Cu–O–Fe directly adsorbs NO and prevents the formation of inactive nitro species, achieving 100% NH_3_ conversion with 99% N_2_ selectivity. Notably, tailoring the d-band of atomic sites and its neighbor O^2–^ will be the key to selective oxidation reactions, which can be extended to other systems. Therefore, the ability to directly probe and design those bridging O^2–^ at the metal/support interface is crucial in catalyst design, mechanism study, and practical applications.

## Supporting Information Available

Additional notes; HAADF–STEM images; XRD patterns; in situ DRIFT spectra; Cu K-edge and Fe K-edge XANES spectra; NAP-NEXAFS spectra; k^3^-weighted EXAFS oscillations; EXAFS fit parameters; DFT calculation results; and NH_3_-SCO performances of catalysts in the literature (PDF)
