# Supplementary material for: Designing Reactive Bridging O2– at the Atomic Cu–O–Fe Site for Selective NH3 Oxidation
Source: ACS Catal. 2022 Nov 29;12(24):15207–17. doi: 10.1021/acscatal.2c04863 (PMC9764355; doi:10.1021/acscatal.2c04863)
Supplement: Supplementary file 1 — cs2c04863_si_001.pdf [file cs2c04863_si_001.pdf]

Supporting Information

# Design reactive bridging $O^{2-}$ at atomic Cu-O-Fe site for selective $NH_3$ oxidation

Xuze Guan,[1] Rong Han,[2] Hiroyuki Asakura,[3,4] Zhipeng Wang,[1] Siyuan Xu,[2] Bolun Wang,[1] Liqun Kang,[1] Yiyun Liu,[1] Sushila Marlow,[1] Tsunehiro Tanaka,[4] Yuzheng Guo,[2]* Feng Ryan Wang[1]*

1. Department of Chemical Engineering, University College London, Roberts Building, Torrington Place, London WC1E 7JE, UK

2. School of Electrical Engineering and Automation, Wuhan University, Wuhan, China

3. Functional Materials Lab, Faculty of Science and Engineering, Kindai University 3-4-1, Kowakae, Higashi-Osaka, Osaka, 577-8502, Japan

4. Department of Molecular Engineering, Graduate School of Engineering, Kyoto University, Kyotodaigaku Katsura, Nishikyo-ku, Kyoto 615-8510, Japan

*Email: ryan.wang@ucl.ac.uk; yguo@whu.edu.cn.

## Supplemental Note 1

To further determine the identity of the second coordination shell, we applied continuous Cauchy wavelet analysis (CCWT) to the k2-weighted EXAFS spectra, since CCWT has proven to be a very useful tool for obtaining structural information from complex systems. The EXAFS spectra can be displayed in a three-dimensional graph: wavevector (k), radial function distribution without phase correction (R) and modulus of the wavelet transform. The position in k-space is proportional to the backscattering factor, so atoms with different Z-numbers will appear in different positions in k-space. the Cu-Cu(2) scattering can be observed in 20wt%CuO-$Fe_2O_3$ at both higher R and k (Figure S6). Therefore, the EXAFS and STEM data converge to the conclusion that the majority of the Cu species in 1wt%CuO-$Fe_2O_3$ are single sites.

## Supplemental Note 2

The relative energy levels of Fe-O-Fe and Fe-O-Cu in figure 5d is deduced from the observed O K edge NEXAFS spectra of pure $Fe_2O_3$ and CuO-$Fe_2O_3$.

First, the Auger electron yield (AEY) mode of NEXASF is an averaging technique that probes the O on surface. For CuO-$Fe_2O_3$, the spectra contain mainly Fe-O-Fe but with trace Fe-O-Cu at the oxidative state.

Second, we use $NH_3$ to reduce CuO-$Fe_2O_3$ at 573 K. Cu is reduced to $Cu^+$ but Fe remains at $Fe^{3+}$. Therefore, only the Fe-O-Cu bond is broken with $NH_3$. The difference in the CuO-$Fe_2O_3$ O K edge spectra between oxidized state and $NH_3$ reduced state comes from the Fe-O-Cu only, which is in Figure 3h.

Third, comparing this Fe-O-Cu spectrum (Figure 3h) with the Fe-O-Fe (Figure 3e, contains mainly Fe-O-Fe but with trace Fe-O-Cu as indicated above) reveals the shift of O(2p) to metal (3d) and to metal (4s, 4p) towards low energy. This means that metal (3d) and metal (4s, 4p) in Fe-O-Cu have a lower energy than that in Fe-O-Fe (Figure 3g).

## Supplemental Figures

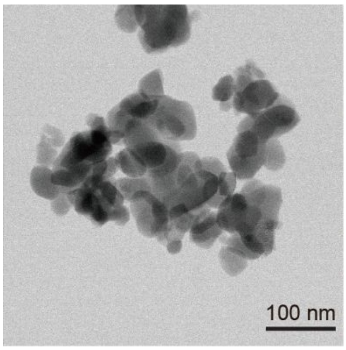


**Figure S1.** Bright field-scanning transmission electron microscopy (BF-STEM) images of pure metal oxides. BF-STEM images of $Fe_2O_3$.

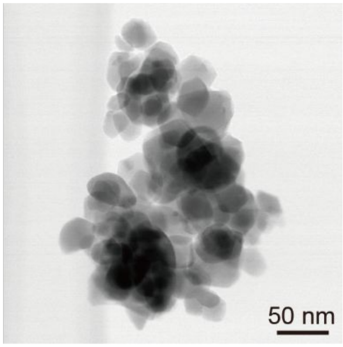


**Figure S2.** BF-STEM images of CuO-$Fe_2O_3$. BF-STEM images of CuO-$Fe_2O_3$ with 1wt% Cu loading.

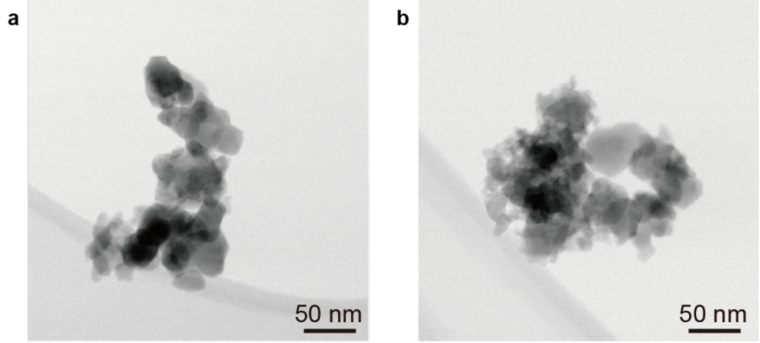


**Figure S3.** BF-STEM images of CuO-$Fe_2O_3$. BF-STEM images of CuO-$Fe_2O_3$ with a) 5wt% and b) 20wt% Cu loading.

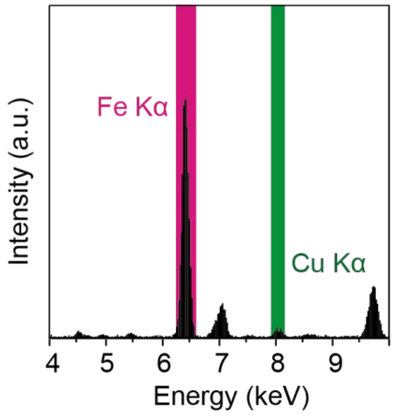


**Figure S4.** Energy dispersive X-ray spectrum (EDX) spectra of CuO-$Fe_2O_3$ with 1wt% Cu loading. Collected from same areas in Figure 1b. Peaks in the pink region and green region correspond to Fe Kα, Cu Kα, respectively.

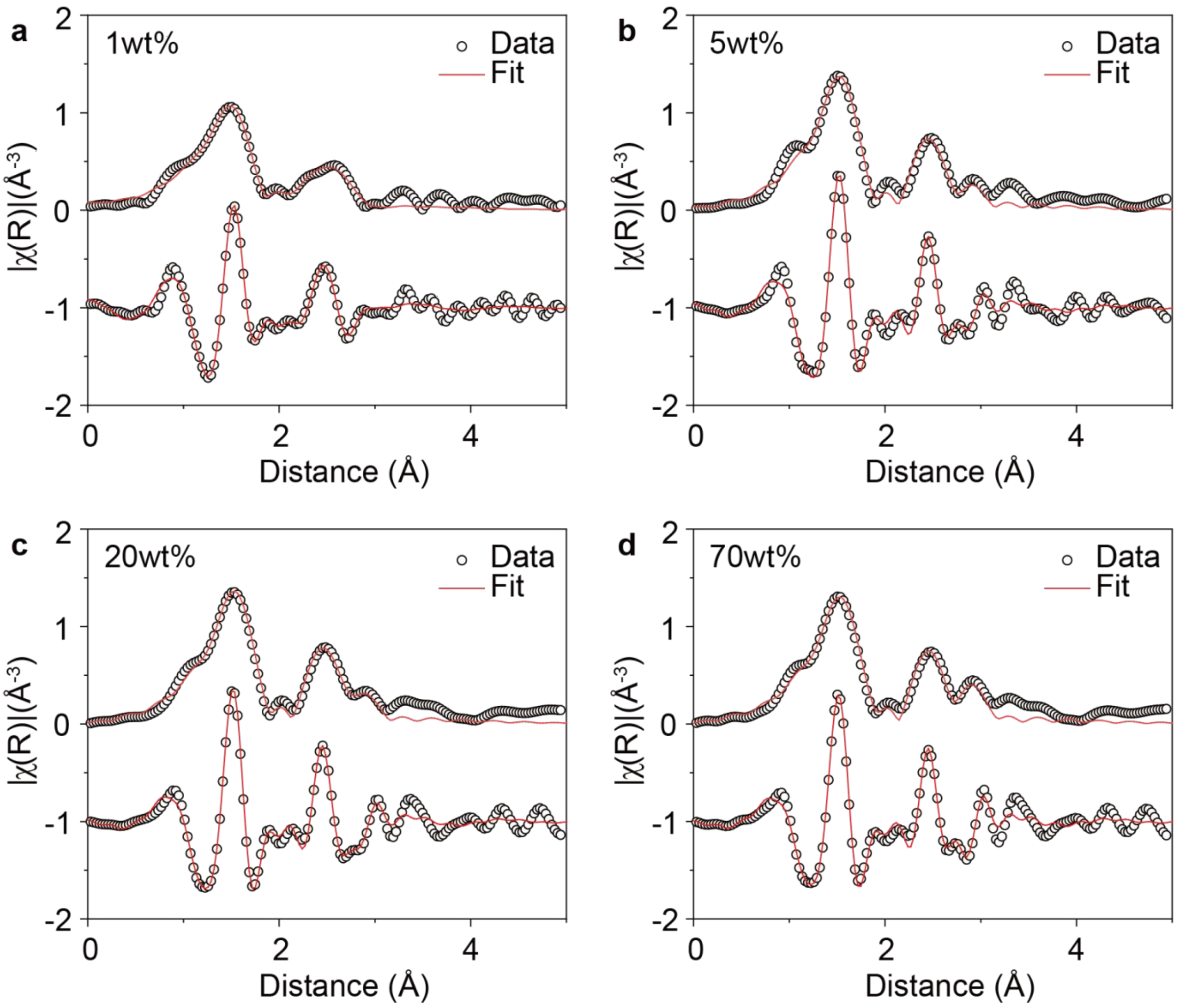


**Figure S5.** EXAFS fitting results of CuO-$Fe_2O_3$ catalysts at different loadings. The $k^2$-weighted Fourier Transform EXAFS data are shown together with fitting results (red curves).

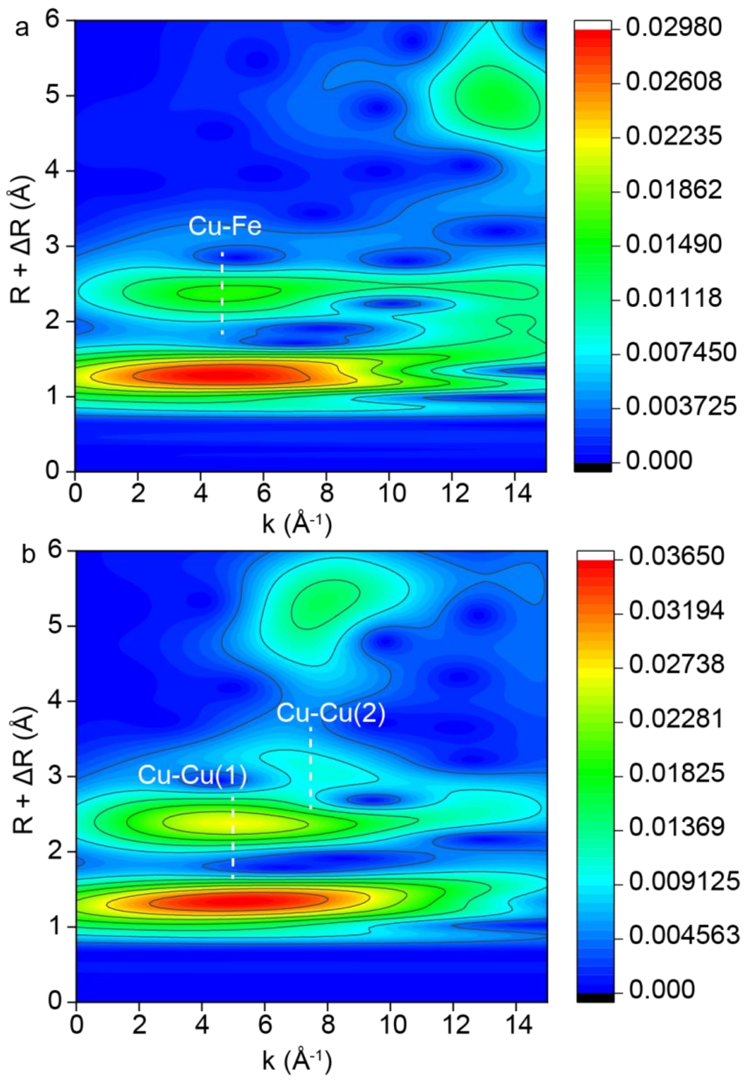


**Figure S6.** CCWT modulus calculated from Cu K edge EXAFS from a) 1wt%CuO-$Fe_2O_3$ and b) 20wt%CuO-$Fe_2O_3$.

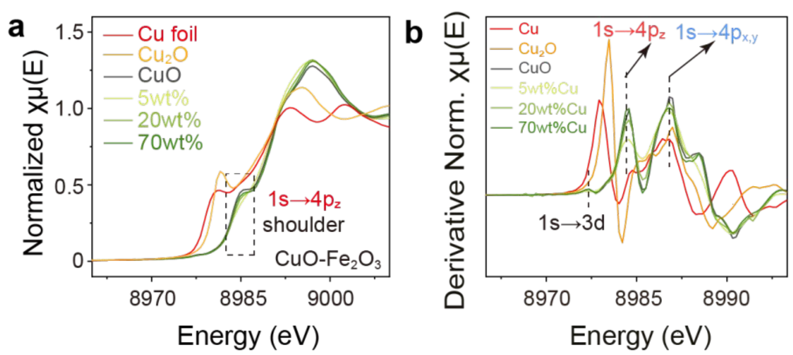


**Figure S7.** a) Cu K edge XANES of CuO-$Fe_2O_3$ with various Cu loading and Cu, $Cu_2O$, CuO standards. b) First derivative of Cu K-edge XANES spectra of CuO-$Fe_2O_3$ with various Cu loading and Cu, $Cu_2O$, CuO standards.

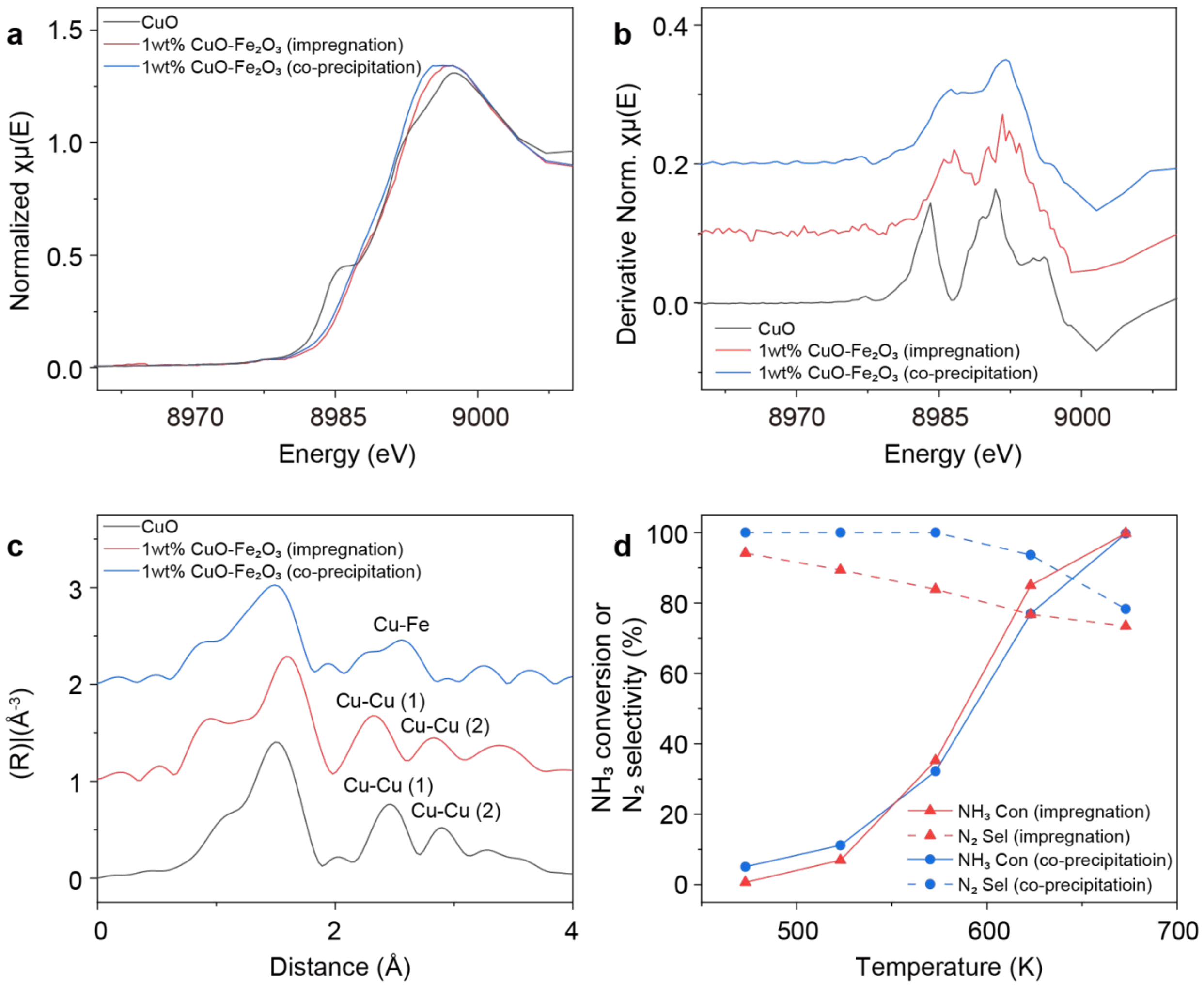


**Figure S8.** XAFS results and catalytic performance of 1wt% $CuO\text{-}Fe_2O_3$ catalysts with different preparation methods. a) Cu K edge X-ray Absorption Near Edge Structure (XANES) of 1wt% $CuO\text{-}Fe_2O_3$ (co-precipitation, blue), 1wt% $CuO\text{-}Fe_2O_3$ (impregnation, red) and CuO (black). b) First derivatives of the XANES spectra. c) $NH_3$ conversion (solid) and $N_2$ selectivity (dash) as the function of temperature for 1wt% $CuO\text{-}Fe_2O_3$ (co-precipitation, blue) and 1wt% $CuO\text{-}Fe_2O_3$ (impregnation, red).

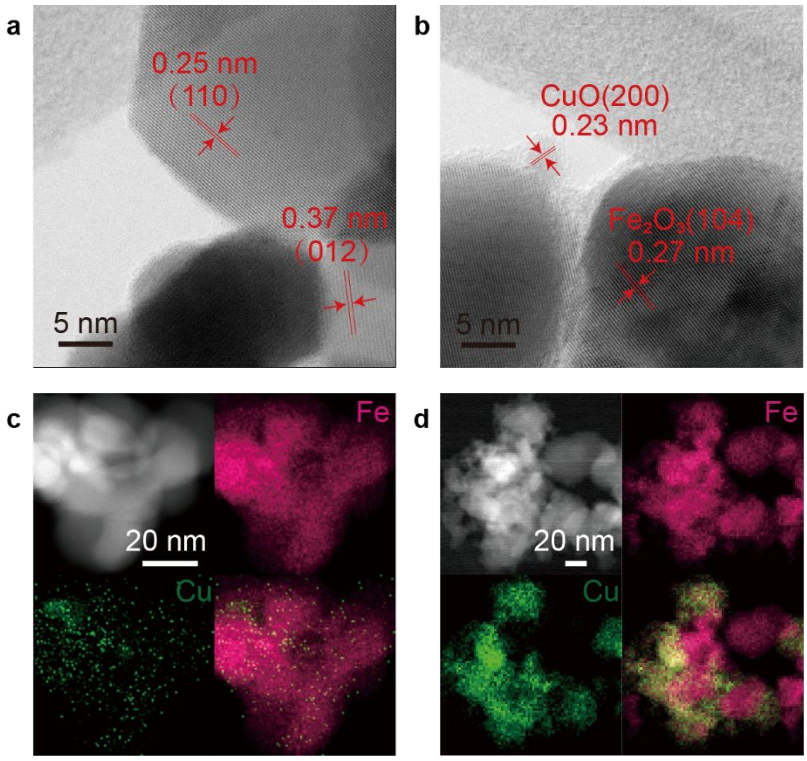


**Figure S9.** Distribution and structure of Cu(II) cluster. a,b) BF-STEM image for CuO-$Fe_2O_3$ with a) 5wt% Cu; b) 20wt% Cu. c,d) EDX mapping of CuO-$Fe_2O_3$ with d) 5wt% Cu and e) 20wt% Cu.

Increasing the Cu loading to 5wt% over $Fe_2O_3$ results in the aggregation of Cu (Figure S11d). At this loading, the lattice fringes of the CuO (200) facet are observed for a 4 nm CuO particle that is interconnected with $Fe_2O_3$ particles (Figure S11b). XRD patterns confirm the crystallinity of $Fe_2O_3$ (Figure S9). The diffraction features of CuO can be observed with 20wt% CuO-$Fe_2O_3$, which is in good agreement with BF-STEM image.For CuO-$Fe_2O_3$ catalysts with Cu loading over 5wt%, EXAFS shows that Cu-Cu scattering occurs at 2.90 ± 0.02Å (Figure S9, S10 and Table S5, S6), corresponding well to CuO standard crystals.

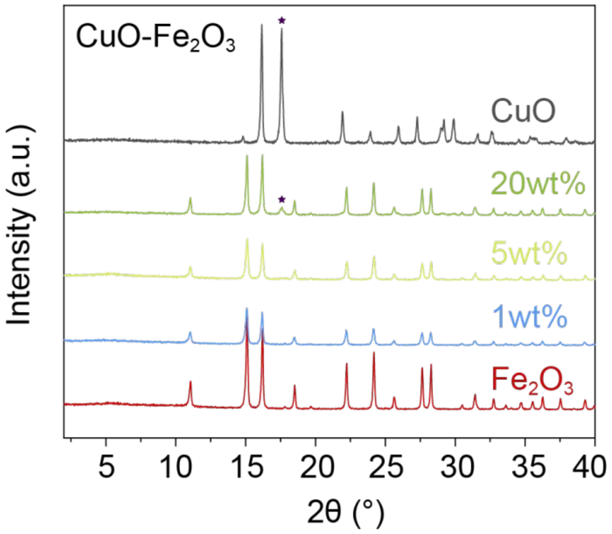


**Figure S10.** X-ray diffraction (XRD) patterns of $CuO$-$Fe_2O_3$ with various Cu loading. XRD of $CuO$-$Fe_2O_3$ with 0wt%, 1wt%, 5wt%, 20wt%, and 100wt% Cu loading.

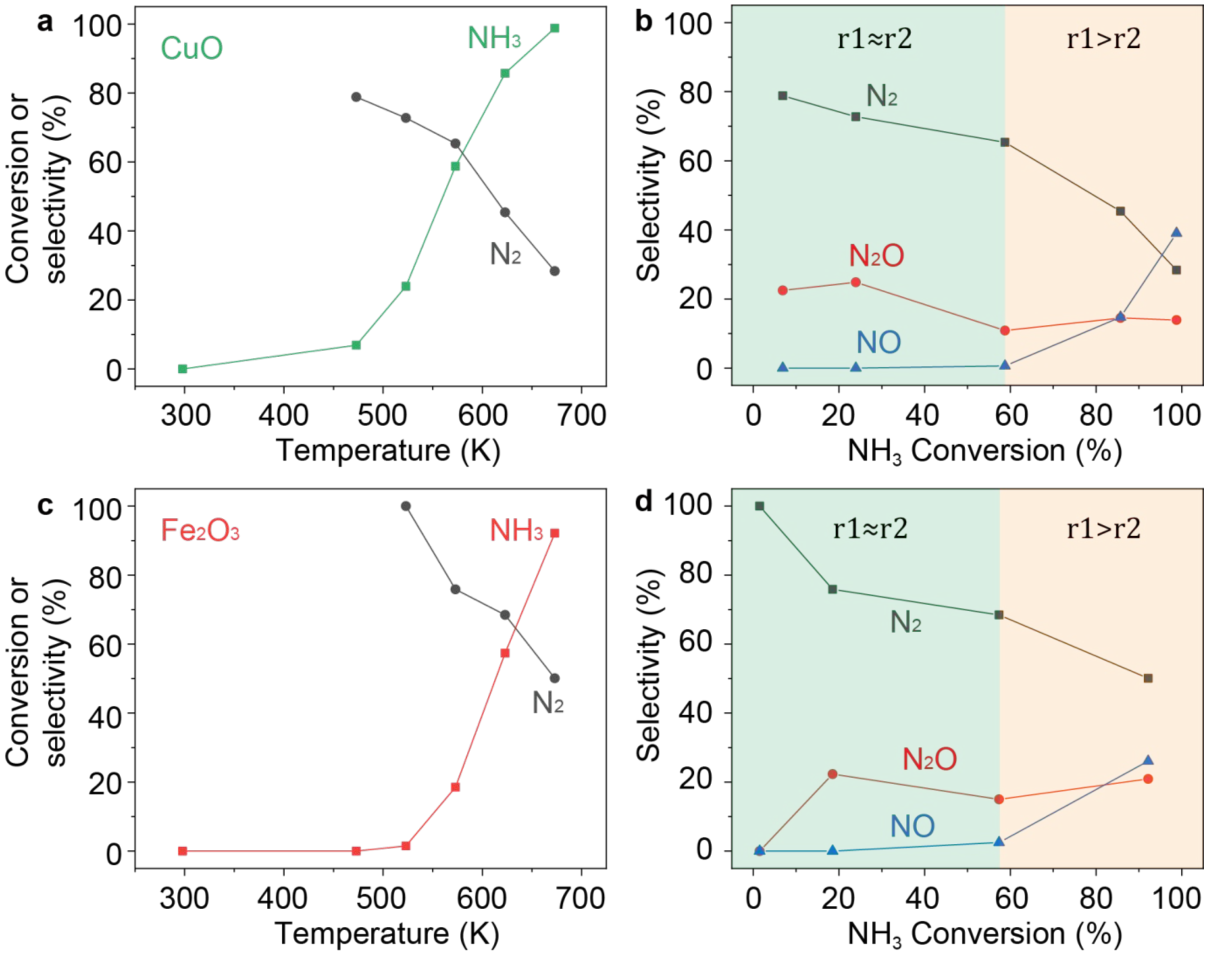


**Figure S11.** Catalytic performance of pure oxides. a) $NH_3$ conversion (green) and $N_2$ selectivity (black) as the function of temperature for pure CuO catalyst; b) Corresponded $N_2$ (black), $N_2O$ (red) and NO (blue) selectivity as the function of conversion for pure CuO catalyst. c) $NH_3$ conversion (red) and $N_2$ selectivity (black) as the function of temperature for pure $Fe_2O_3$ catalyst; d) Corresponded $N_2$ (black), $N_2O$ (red) and NO (blue) selectivity as the function of conversion for pure $Fe_2O_3$ catalyst.

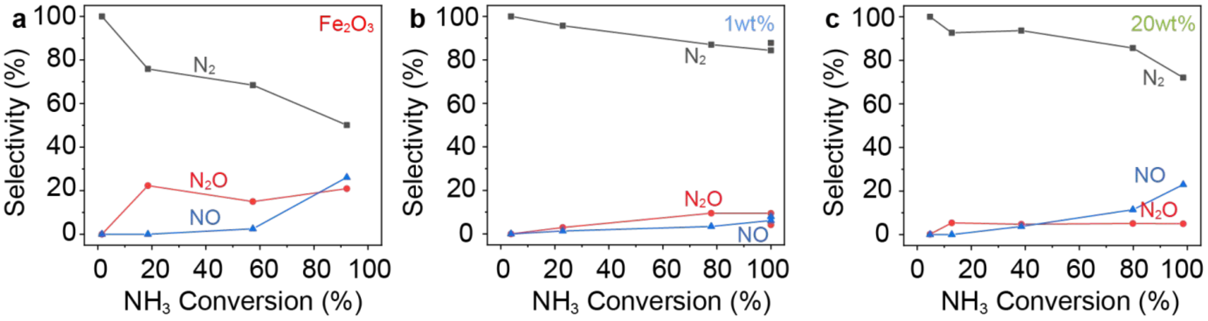


**Figure S12.** Catalytic performance of CuO-$Fe_2O_3$ catalysts in $NH_3$ oxidation. $N_2$ (black), $N_2O$ (red) and NO (blue) selectivity as the function of conversion of a) pure $Fe_2O_3$ catalyst; b) CuO-$Fe_2O_3$ catalyst with 1wt% Cu; c) CuO-$Fe_2O_3$ catalyst with 20wt% Cu. Reaction condition: 50mg catalyst, 5000ppm $NH_3$, 5% $O_2$ balanced in He, gas flow: 100mL/min, WHSV=600 $ml_{NH3}\cdot h^{-1}\cdot g^{-1}$.

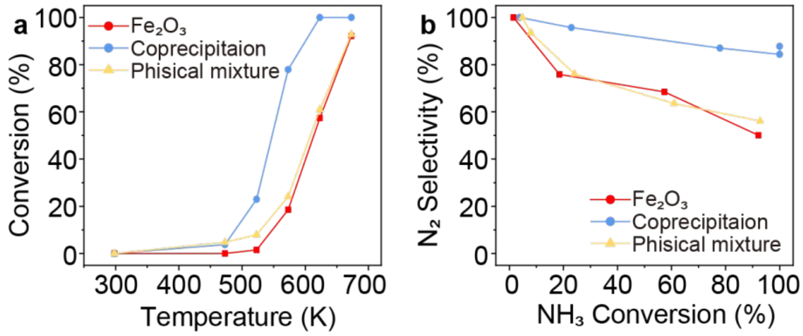


**Figure S13.** Catalytic performance of physically mixed 1wt% CuO + 99wt% $Fe_2O_3$. a) $NH_3$ conversion as the function of temperature; b) $N_2$ selectivity as the function of conversion.

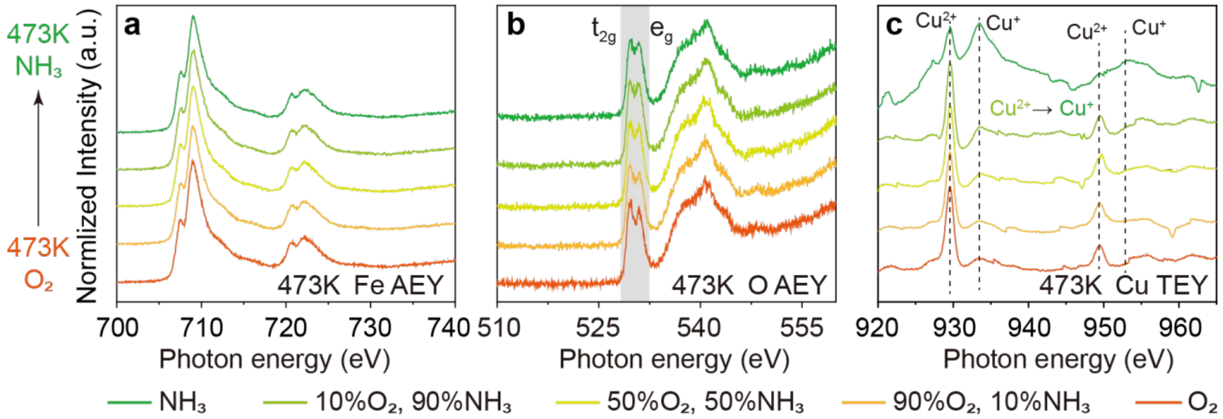


**Figure S14.** NEXAFS spectra of 1wt% $CuO$-$Fe_2O_3$ under various gas conditions at 473 K. a) Fe L-edge (AEY mode), b) O K-edge (AEY mode) and c) Cu L-edge (TEY mode) of 1wt% $CuO$-$Fe_2O_3$ at 473 K.

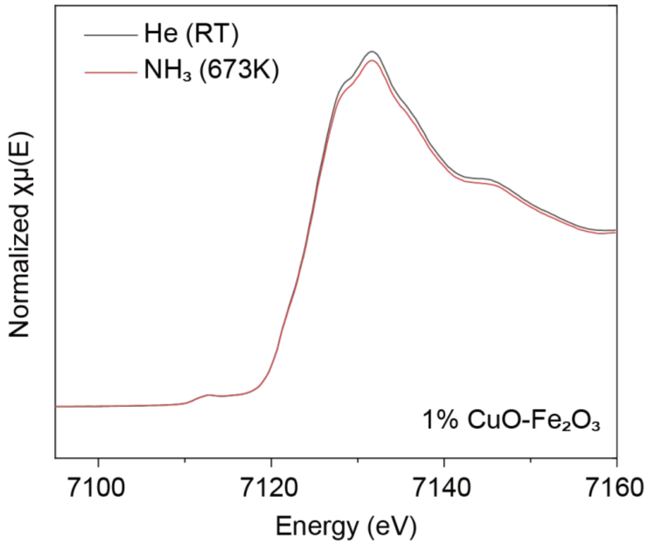


**Figure S15.** *In situ* XANES of Fe K edge of 1wt% CuO-$Fe_2O_3$. Fe K edge XANES of 1wt% CuO-$Fe_2O_3$ under He at RT and $NH_3$ at 673K.

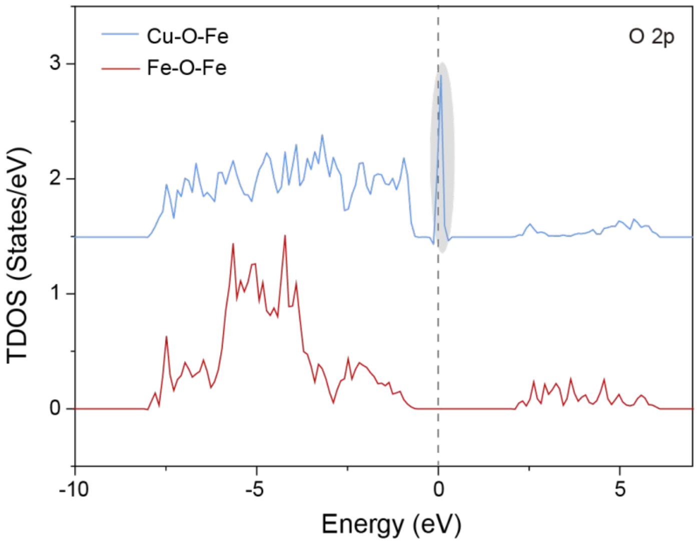


**Figure S16.** The PDOS of O-p orbit in Cu-O-Fe and Fe-O-Fe in the Cu single site over $Fe_2O_3$.

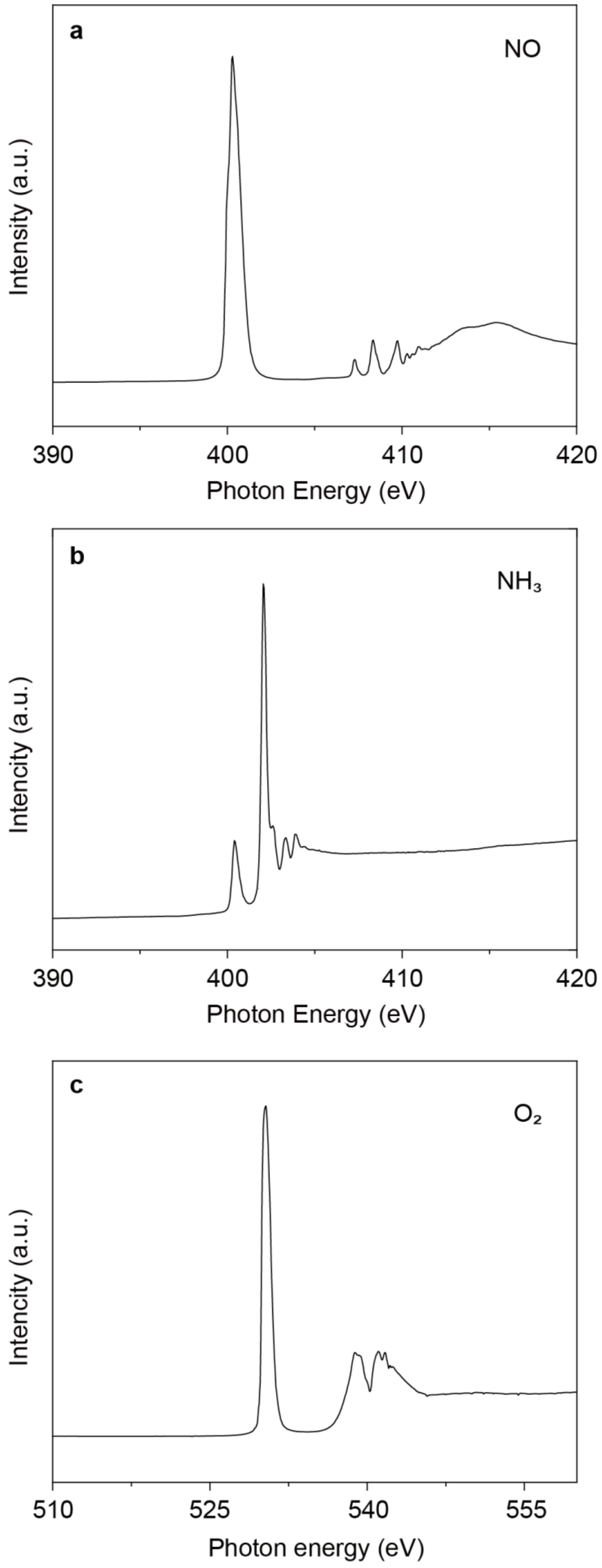


**Figure S17.** NAP-NEXAFS of gaseous NO, $NH_3$ and $O_2$. a,b) N K edge NAP-NEXAFS spectra (TEY) of a) NO and b) $NH_3$. c) O K edge NAP-NEXAFS spectra (TEY) of c $O_2$. The TEY mode measures the gases in the system.

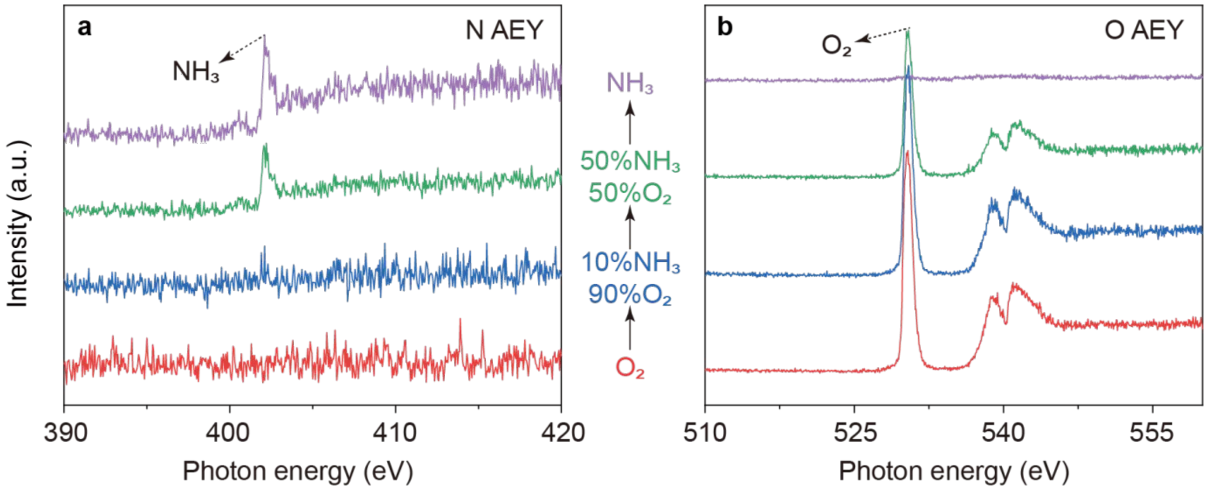


**Figure S18.** NAP-NEXAFS of competitive adsorption of $NH_3$ and $O_2$ over 1wt% CuO-$Fe_2O_3$. a) N K-edges and b) O K-edges NEXAFS spectra (AEY mode) of 1wt% CuO-$Fe_2O_3$ under various gas conditions (0.3 mbar) at 298K. The AEY mode measures the surface adsorbed species. The surface absorbed $NH_3$ and $O_2$ have similar features to the $NH_3$ and $O_2$ in the gas phase (Figure S16).

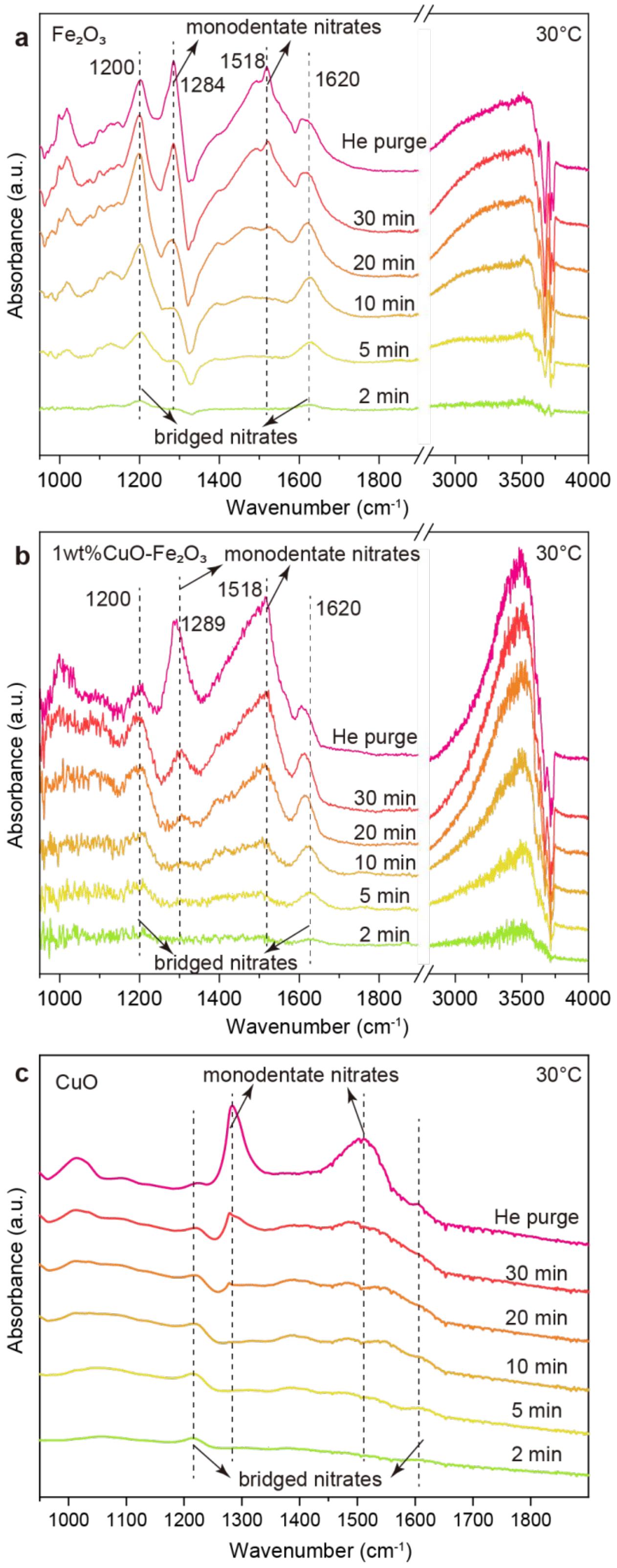


**Figure S19.** *In situ* DRIFTS study of NO absorption over $Fe_2O_3$, 1wt% CuO-$Fe_2O_3$ and CuO. *In situ* DRIFTS spectra of NO adsorption on a) $Fe_2O_3$, b) 1wt% CuO-$Fe_2O_3$ and c) CuO as a function of time. The catalyst was exposed to a flow of 500 ppm NO for 30 min at 30 ℃ and then purged with He.

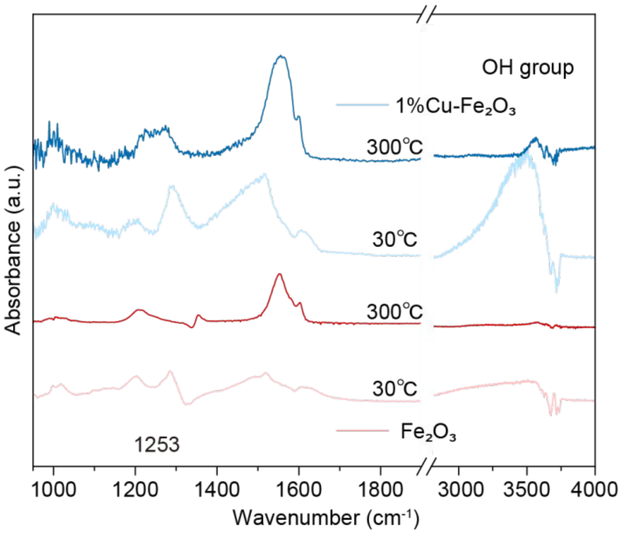


**Figure S20.** *In situ* DRIFTS spectra of NO desorption on 1wt% CuO-$Fe_2O_3$ (blue) and $Fe_2O_3$ (red) as a function of temperature after the catalyst was exposed to a flow of 500 ppm NO for 30 min at 30 ℃.

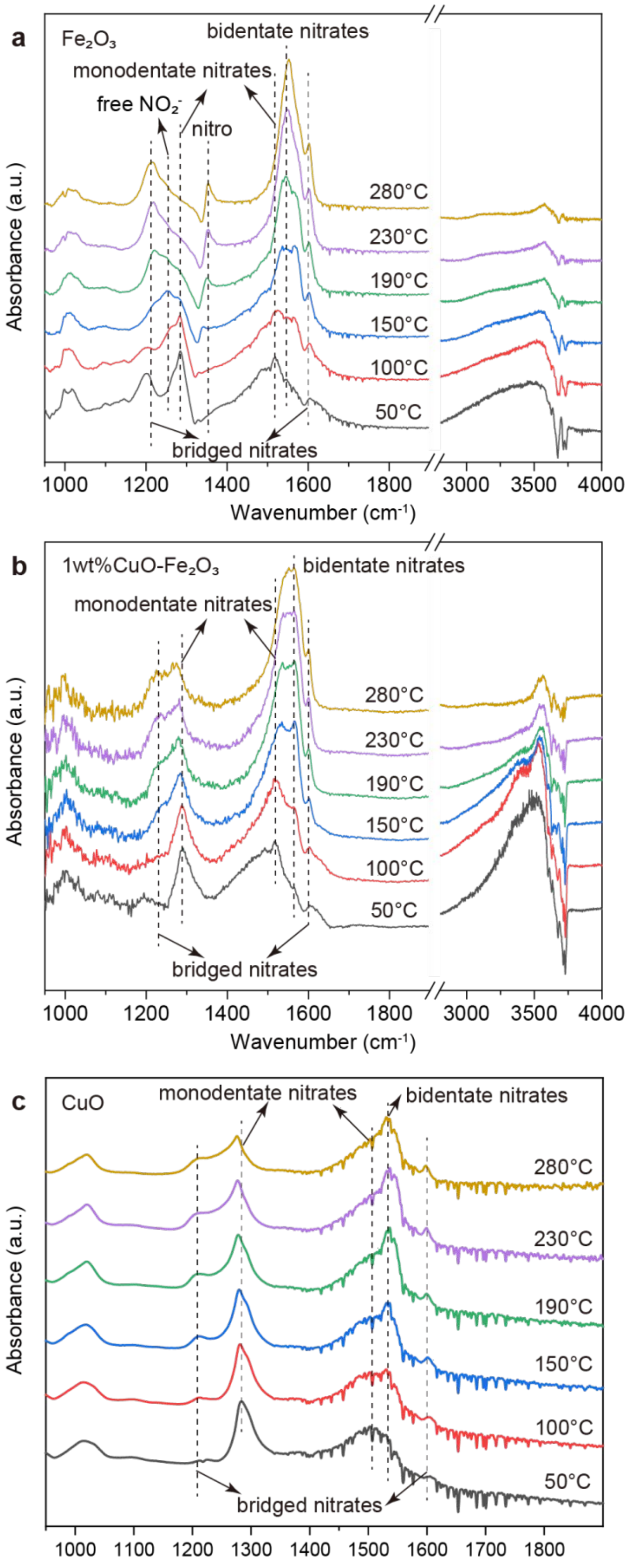


**Figure S21.** *In situ* DRIFTS study of NO desorption over $Fe_2O_3$, 1wt% CuO-$Fe_2O_3$ and CuO. *In situ* DRIFTS spectra of NO desorption in He on a) $Fe_2O_3$, b) 1wt% CuO-$Fe_2O_3$ and c) CuO as a function of temperature after the catalyst was exposed to a flow of 500 ppm NO for 30 min at 30 ℃.

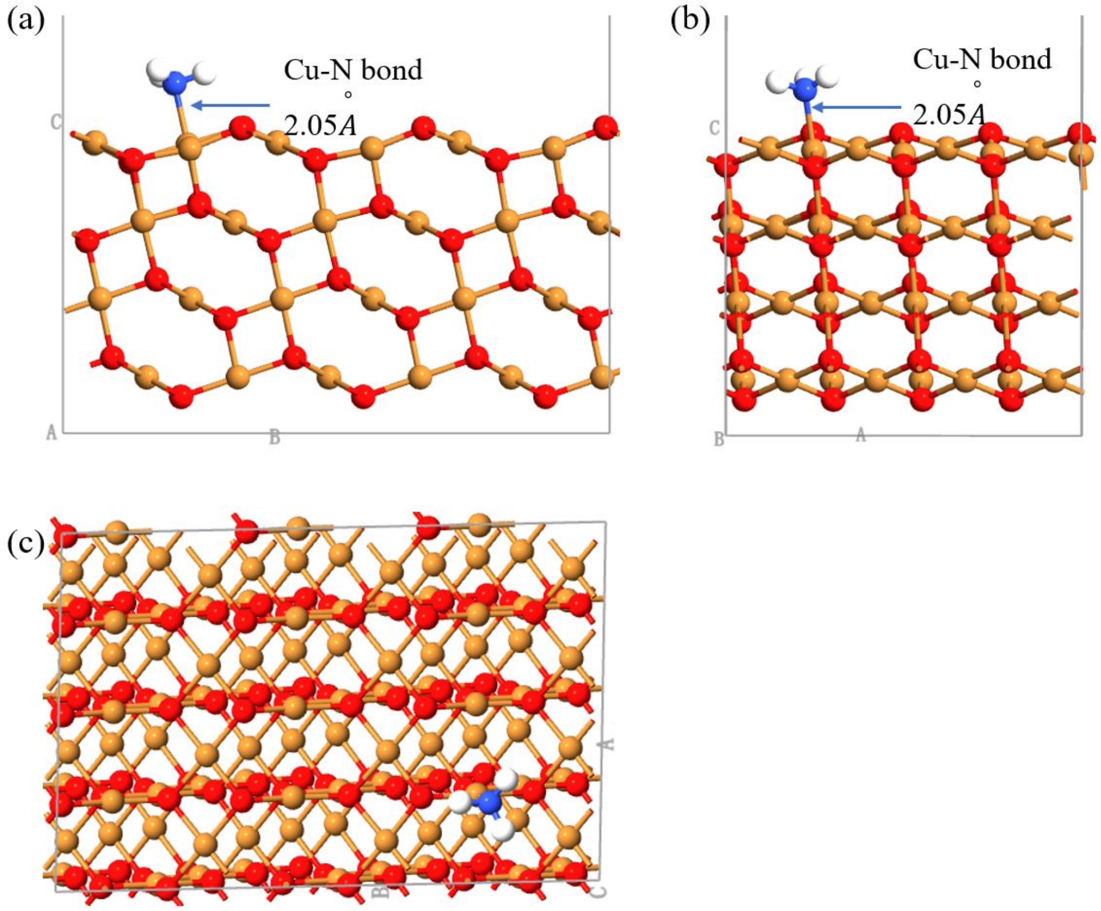


**Figure S22.** Density Functional Theory (DFT) calculation of $NH_3$ adsorption on CuO (111) surface. The a) front, b) side and c) overview of the $NH_3$ molecule adsorption on CuO (111) surface. Cu atoms are yellow, O atoms are red, H atoms are white and N atoms are blue.

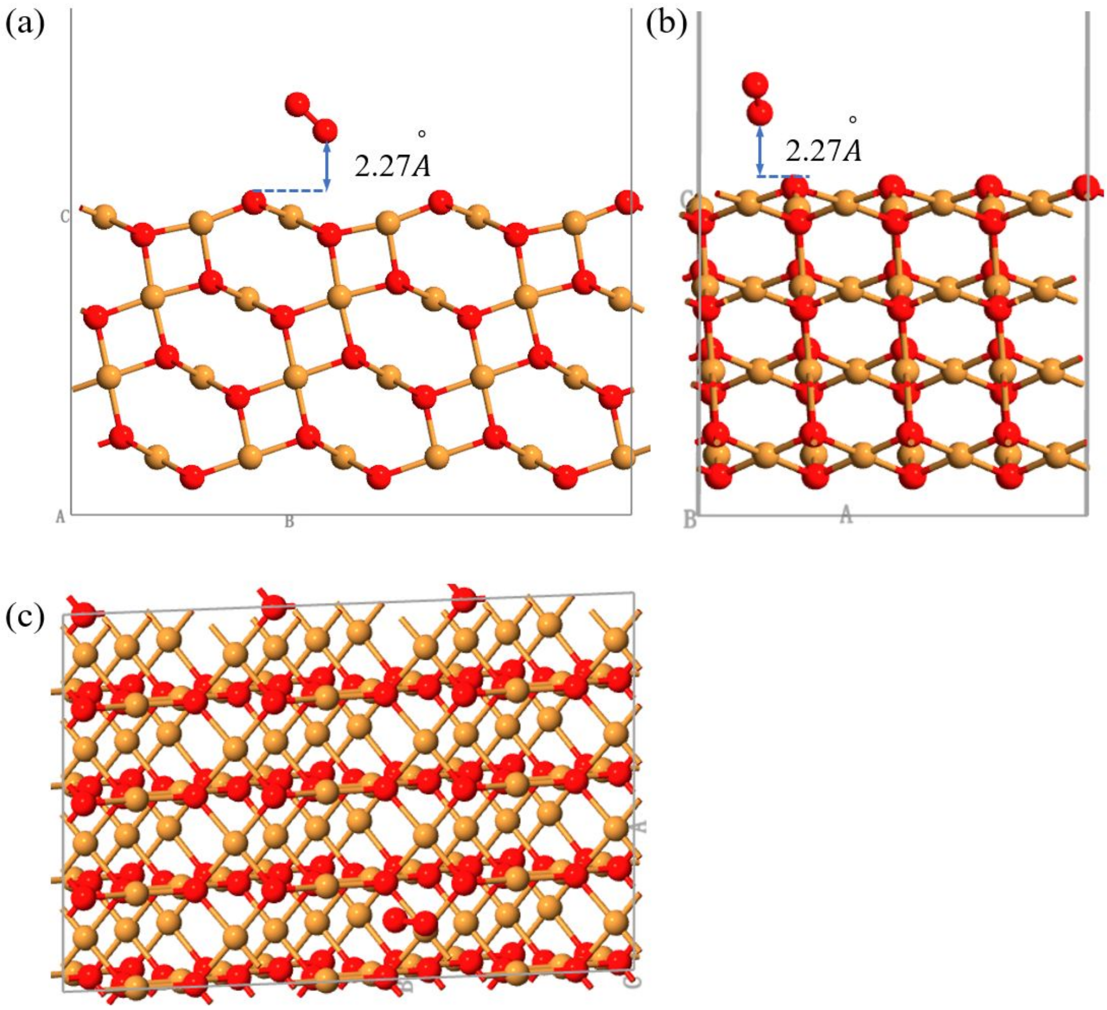


**Figure S23.** DFT calculation of $O_2$ adsorption on CuO (111) surface. The a) front, b) side and c) overview of the $O_2$ molecule adsorption on CuO (111) surface. Cu atoms are yellow and O atoms are red.

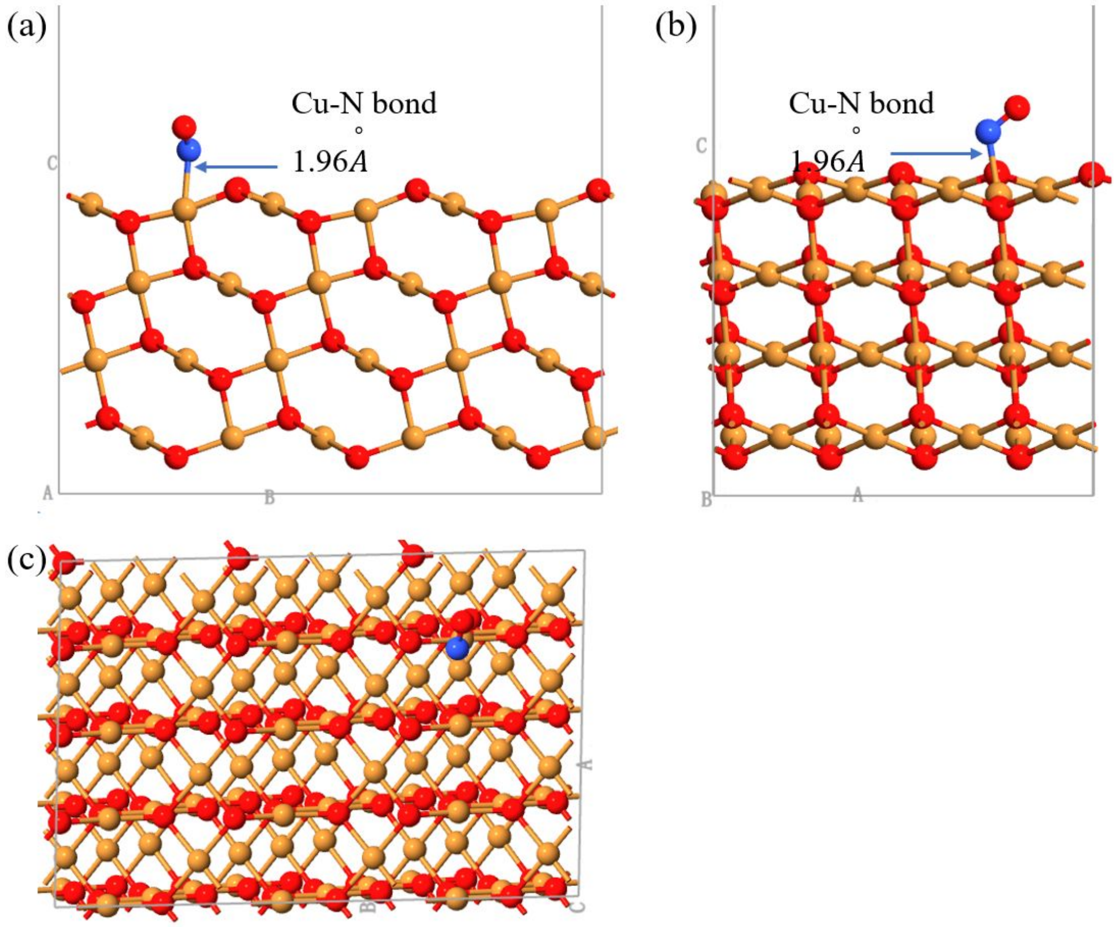


**Figure S24.** DFT calculation of NO adsorption on CuO (111) surface. The a) front, b) side and c) overview of the NO molecule adsorption on CuO (111) surface. Cu atoms are yellow, O atoms are red and N atoms are blue.

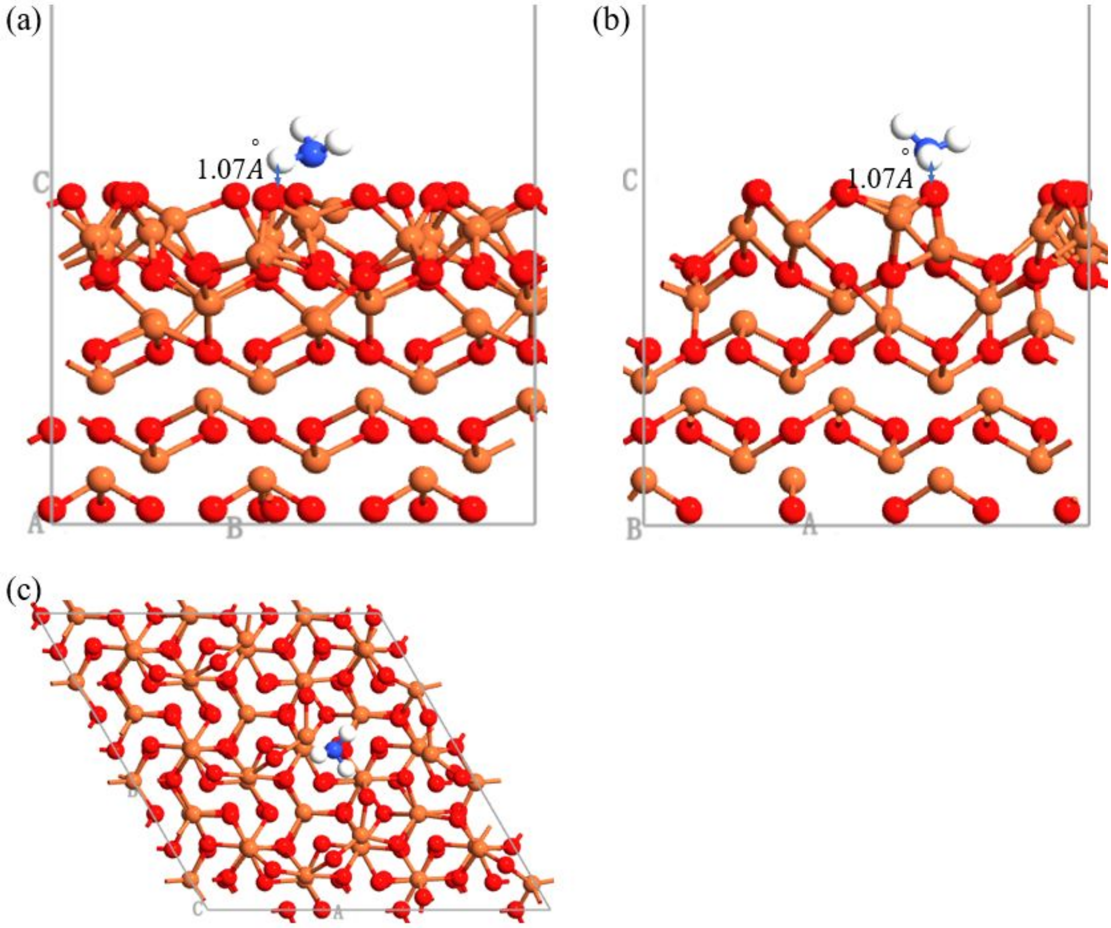


**Figure S25.** DFT calculation of $NH_3$ adsorption on $Fe_2O_3$ (0001) surface. The a) front, b) side and c) overview of the $NH_3$ molecule adsorption on $Fe_2O_3$ (0001) surface. Fe atoms are orange, O atoms are red, H atoms are white and N atoms are blue.

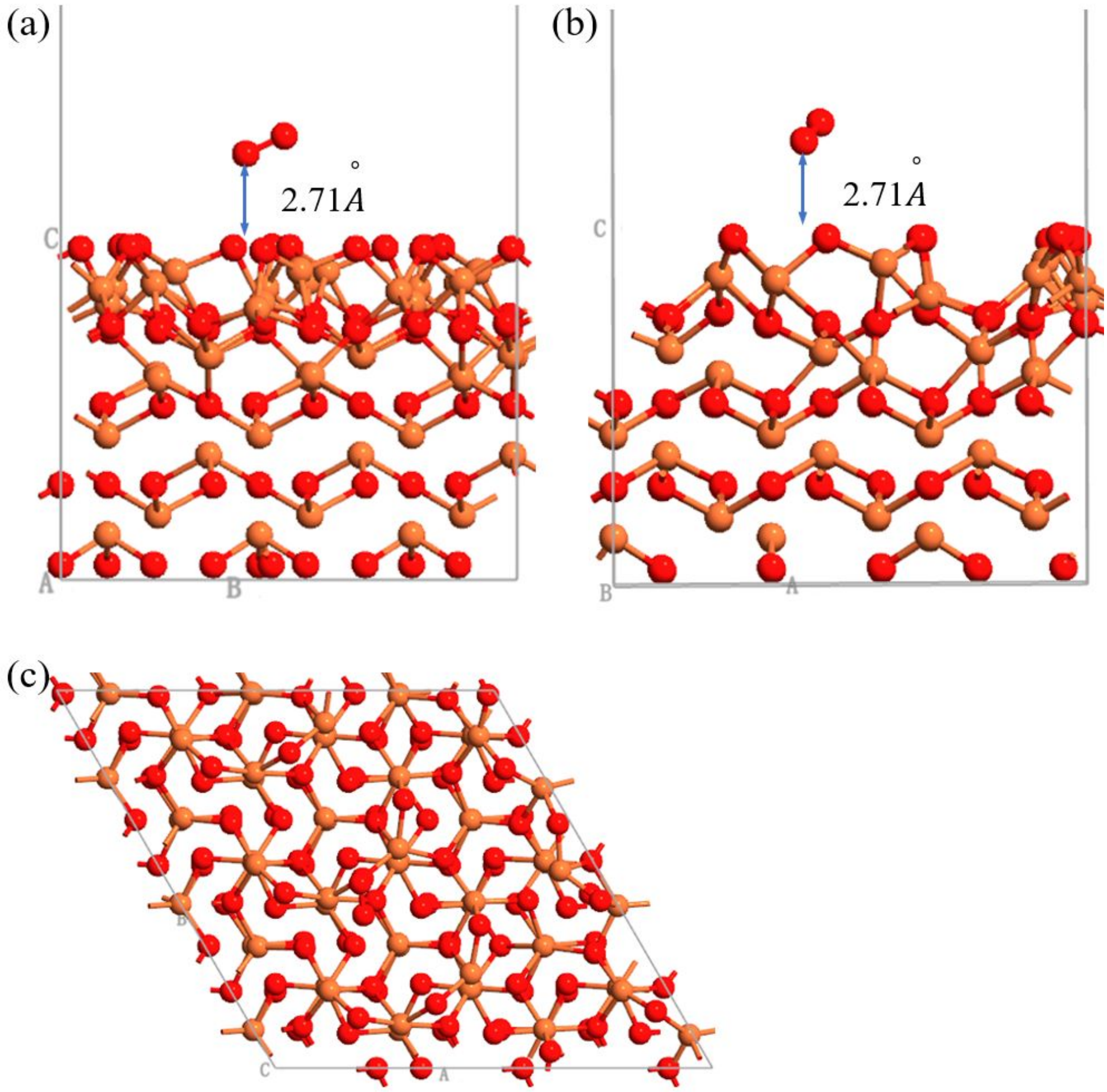


**Figure S26.** DFT calculation of $O_2$ adsorption on $Fe_2O_3$ (0001) surface. The a) front, b) side and c) overview of the $O_2$ molecule adsorption on $Fe_2O_3$ (0001) surface. Fe atoms are orange and O atoms are red.

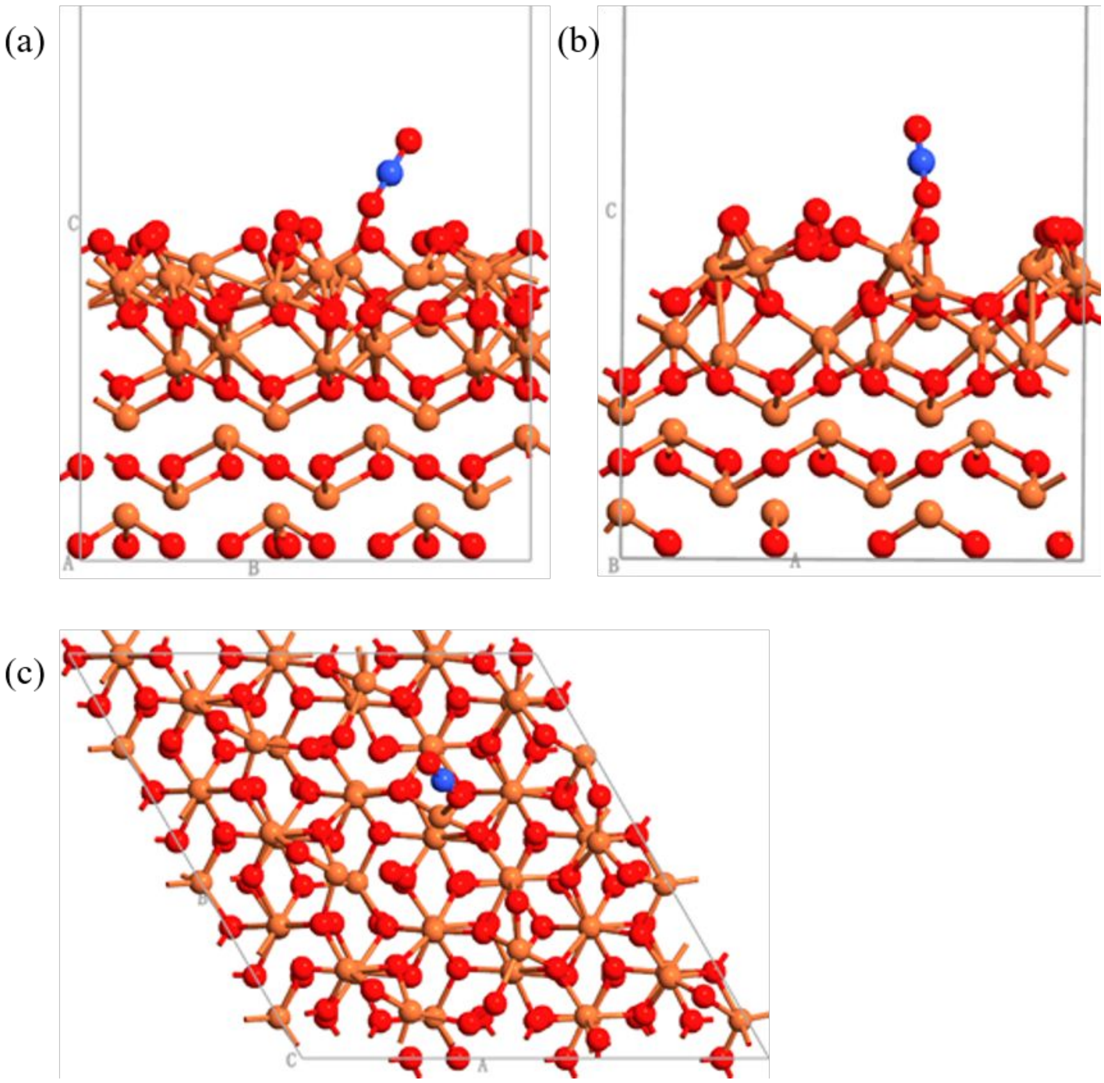

**Figure S27.** DFT calculation of NO adsorption on $Fe_2O_3$ (0001) surface. The a) front, b) side and c) overview of the NO molecule adsorption on $Fe_2O_3$ (0001) surface. Fe atoms are orange, O atoms are red and N atoms are blue.

## Supplemental Tables

**Table S1** EXAFS fitting results of CuO-$Fe_2O_3$ catalysts.

| Sample | Scattering | C.N. | d (Å) | $\sigma^2$ | $E_0$ (eV) |
|---|---|---|---|---|---|
| Cu foil STD | Cu-Cu | 12 | 2.56 | | |
| $Cu_2O$ STD | Cu-O | 2 | 1.85 | | |
| | Cu-Cu | 12 | 3.01 | | |
| CuO STD | Cu-O | 4 | 1.95 | | |
| | Cu-Cu | 4 | 2.88 | | |
| | | 4 | 3.07 | | |
| | | 2 | 3.16 | | |
| $Fe_2O_3$ STD | Fe-O | 3 | 1.91 | | |
| | | 3 | 2.11 | | |
| | Fe-Fe | 1 | 2.73 | | |
| | | 3 | 2.92 | | |
| 1wt% CuO-$Fe_2O_3$ | Cu-O | 3.61 ± 0.21 | 1.94 ± 0.01 | 0.006 ± 0.001 | -4.71 ± 0.60 |
| | Cu-Fe | 2.66 ± 0.58 | 2.94 ± 0.01 | 0.015 ± 0.003 | |
| 5wt% CuO-$Fe_2O_3$ | Cu-O | 3.72 ± 0.41 | 1.95 ± 0.01 | 0.004 ± 0.001 | -1.23 ± 1.22 |
| | Cu-Cu (1) | 3.98 ± 1.57 | 2.90 ± 0.02 | 0.008 ± 0.003 | |
| | Cu-Cu (2) | 2.95 ± 1.28 | 3.08 ± 0.03 | | |
| 20wt% CuO-$Fe_2O_3$ | Cu-O | 3.69 ± 0.30 | 1.96 ± 0.01 | 0.004 ± 0.001 | -0.11 ± 0.88 |
| | Cu-Cu (1) | 4.74 ± 1.26 | 2.90 ± 0.01 | 0.008 ± 0.002 | |
| | Cu-Cu (2) | 3.83 ± 1.03 | 3.08 ± 0.02 | | |
| 70wt% CuO-$Fe_2O_3$ | Cu-O | 3.54 ± 0.38 | 1.96 ± 0.01 | 0.004 ± 0.001 | 0.19 ± 1.19 |
| | Cu-Cu (1) | 4.42 ± 1.34 | 2.90 ± 0.01 | 0.007 ± 0.002 | |
| | Cu-Cu (2) | 4.02 ± 1.33 | 3.09 ± 0.02 | | |

**Table S2** Peak position of XANES spectra of CuO-$Fe_2O_3$.

| Sample | Absorption peak position (keV) | | | | | White line position (keV) |
|---|---|---|---|---|---|---|
| | 1s to 3*d* [a] | 1s to 4$p_z$ [a] | 1s to 4$p_{xy}$ [a] | 1s to 4$p_z$ [b] | 1s to 4*p* [b] | |
| Cu STD | | 8979.0 | | | | 8993.6 |
| $Cu_2O$ STD | | 8980.6 | | | 8981.7 | 8995.6 |
| CuO STD | 8977.3 | 8984.0 | 8990.8 | 8986.0 | | 8997.5 |
| 1wt% | 8976.2 | 8986.7 | 8991.9 | - | | 8996.4 |
| 5wt% | 8976.7 | 8984.0 | 8990.8 | 8986.2 | | 8997.0 |
| 20wt% | 8977.3 | 8984.0 | 8990.8 | 8986.0 | | 8997.5 |
| 70wt% | 8977.3 | 8984.0 | 8990.8 | 8986.0 | | 8997.5 |

[a] Peak position in first derivative XANES.
[b] Peak position in XANES.

**Table S3** Bader charge of 1wt% CuO-$Fe_2O_3$.

| 1wt% CuO-$Fe_2O_3$ | | | | | |
|---|---|---|---|---|---|
| | Cu | $O_1$ | $O_2$ | $O_3$ | $O_4$ |
| Bader charge (e) | -0.99 | 0.94 | 0.93 | 0.93 | 0.66 |
| Bond length (Å) | - | 1.94 | 1.97 | 2.07 | 1.87 |

**Table S4** Bader charge of pure CuO.

| CuO | | | | | |
|---|---|---|---|---|---|
| | Cu | $O_1$ | $O_2$ | $O_3$ | $O_4$ |
| Bader charge (e) | -0.89 | 0.86 | 0.88 | 0.87 | 0.85 |
| Bond length (Å) | - | 1.93 | 1.93 | 1.93 | 1.93 |

**Table S5** Review of catalytic performance of different catalysts for $NH_3$-SCO.

| Catalysts | T (K) | $NH_3$ conversion (%) | $N_2$ selectivity (%) | WHSV ($ml_{NH3}\cdot h^{-1}\cdot g^{-1}$) |
|---|---|---|---|---|
| 1wt%CuO-$Fe_2O_3$ | 573 | 100 | 99 | 120 |
| 1wt%Pt/$Al_2O_3$ | 473 | 91 | 73 | 120 |
| | 573 | 100 | 80 | |
| 10wt%CuO-$Al_2O_3$[3] | 623 | 100 | 93 | 30 |
| 30wt%CuO-$RuO_2$[4] | 483 | 100 | 99 | 75 |
| 1.5%Ag-10% Cu/$Al_2O_3$[5] | 648 | 100 | 94 | 120 |
| **CuO/CNTs** (10 wt.%)[6] | 462 | 100 | 98.7 | 60 |
| 10wt%Cu/$TiO_2$[7] | 523 | 100 | 95 | 60 |

**Table S6** EXAFS fitting results of CuO-$Fe_2O_3$ catalysts under $O_2$ and $NH_3$.

| Sample | Scattering | C.N. | d (Å) | $\sigma^2$ | $E_0$ (eV) |
|---|---|---|---|---|---|
| Cu foil STD | Cu-Cu | 12 | 2.56 | | |
| $Cu_2O$ STD | Cu-O | 2 | 1.85 | | |
| CuO STD | Cu-O | 4 | 1.95 | | |
| 1wt% CuO-$Fe_2O_3$ in $O_2$ at 573 K | Cu-O | 3.28 ± 0.66 | 1.94 ± 0.02 | 0.006 | -7.74 ± 2.68 |
| 1wt% CuO-$Fe_2O_3$ in $NH_3$ at 573 K | Cu-O | 2.40 ± 0.48 | 1.82 ± 0.05 | 0.008 | -0.89 ± 7.14 |

**Table S7** Calculated $NH_3$, NO and $O_2$ adsorption energy on CuO (111) and $Fe_2O_3$ (0001) surfaces.

| Surface | Adsorbent | $E_{surf}$ (eV) | $E_{surf+mol}$ (eV) | $E_{ad}$ (eV) |
|---|---|---|---|---|
| CuO (111) | $NH_3$ | -767.29 | -787.96 | -1.12 |
| | NO | | -780.62 | -1.02 |
| | $O_2$ | | -777.28 | -0.13 |
| $Fe_2O_3$ (0001) | $NH_3$ | -1187.24 | -1209.30 | -1.06 |
| | NO | | -1185.41 | -1.12 |
| | $O_2$ | | -1197.86 | -0.76 |

**Table S8** Bader charge of 1wt% CuO-$Fe_2O_3$ before adsorption of NO and after adsorption of NO.

| Bader charge (e) | Cu | $O_1$ | $O_2$ | $O_3$ | $O_4$ |
|---|---|---|---|---|---|
| Before | -0.99 | 0.94 | 0.93 | 0.93 | 0.66 |
| After | -0.92 | 0.92 | 0.97 | 0.94 | 0.56 |

## References


1. Munoz, M.; Argoul, P.; Farges, F., Continuous Cauchy wavelet transform analyses of EXAFS spectra: A qualitative approach. *American Mineralogist* **2003,** *88* (4), 694-700.
2. Otal, E. H.; Sileo, E.; Aguirre, M. H.; Fabregas, I. O.; Kim, M., Structural characterization and EXAFS wavelet analysis of Yb doped ZnO by wet chemistry route. *Journal of Alloys and Compounds* **2015,** *622*, 115-120.
3. Liang, C. X.; Li, X. Y.; Qu, Z. P.; Tade, M.; Liu, S. M., The role of copper species on Cu/gamma-Al2O3 catalysts for NH3-SCO reaction. *Appl. Surf. Sci.* **2012,** *258* (8), 3738-3743.
4. Cui, X. Z.; Zhou, J.; Ye, Z. Q.; Chen, H. R.; Li, L.; Ruan, M. L.; Shi, J. L., Selective catalytic oxidation of ammonia to nitrogen over mesoporous CuO/RuO2 synthesized by co-nanocasting-replication method. *J. Catal.* **2010,** *270* (2), 310-317.
5. Jablonska, M.; Beale, A. M.; Nocun, M.; Palkovits, R., Ag-Cu based catalysts for the selective ammonia oxidation into nitrogen and water vapour. *Appl. Catal. B-Environ.* **2018,** *232*, 275-287.
6. Song, S. Q.; Jiang, S. J., Selective catalytic oxidation of ammonia to nitrogen over CuO/CNTs: The promoting effect of the defects of CNTs on the catalytic activity and selectivity. *Appl. Catal. B-Environ.* **2012,** *117*, 346-350.
7. He, S. L.; Zhang, C. B.; Yang, M.; Zhang, Y.; Xu, W. Q.; Cao, N.; He, H., Selective catalytic oxidation of ammonia from MAP decomposition. *Sep. Purif. Technol.* **2007,** *58* (1), 173-178.
